# Reversible redox switching of magnetic order and electrical conductivity in a 2D manganese benzoquinoid framework†
†Electronic supplementary information (ESI) available. CCDC 1894228–1894230. For ESI and crystallographic data in CIF or other electronic format see DOI: 10.1039/c9sc00606k


**DOI:** 10.1039/c9sc00606k

**Published:** 2019-03-14

**Authors:** Lujia Liu, Jordan A. DeGayner, Lei Sun, David Z. Zee, T. David Harris

**Affiliations:** a Department of Chemistry , Northwestern University , 2145 Sheridan Road , Evanston , Illinois 60208-3113 , USA . Email: dharris@northwestern.edu

## Abstract

Materials with switchable magnetic and electrical properties may enable future spintronic technologies, and thus hold the potential to revolutionize how information is processed and stored. While reversible switching of magnetic order or electrical conductivity has been independently realized in materials, the ability to simultaneously switch both properties in a single material presents a formidable challenge. Here, we report the 2D manganese benzoquinoid framework (Me_4_N)_2_[MnII2(L^2–^)_3_] (H_2_L = 2,5-dichloro-3,6-dihydroxo-1,4-benzoquinone), as synthesized *via* post-synthetic counterion exchange. This material is paramagnetic above 1.8 K and exhibits an ambient-temperature electrical conductivity of *σ*_295 K_ = 1.14(3) × 10^–13^ S cm^–1^ (*E*_a_ = 0.74(3) eV). Upon soaking in a solution of sodium naphthalenide and 1,2-dihydroacenaphthylene, this compound undergoes a single-crystal-to-single-crystal (SC–SC) reduction to give Na_3_(Me_4_N)_2_[Mn_2_L_3_]. Structural and spectroscopic analyses confirm this reduction to be ligand-based, and as such the anionic framework is formulated as [MnII2(L^3–^˙)_3_]^5–^. Magnetic measurements confirm that this reduced material is a permanent magnet below *T*_c_ = 41 K and exhibits a conductivity value of *σ*_295 K_ = 2.27(1) × 10^–8^ S cm^–1^ (*E*_a_ = 0.489(8) eV), representing a remarkable 200 000-fold increase over the parent material. Finally, soaking the reduced compound in a solution of [Cp_2_Fe]^+^ affords Na(Me_4_N)[MnII2(L^2–^)_3_] *via* a SC–SC process, with magnetic and electrical properties similar to those observed for the original oxidized material. Taken together, these results highlight the ability of metal benzoquinoid frameworks to undergo reversible, simultaneous redox switching of magnetic order and electrical conductivity.

## Abstract 2

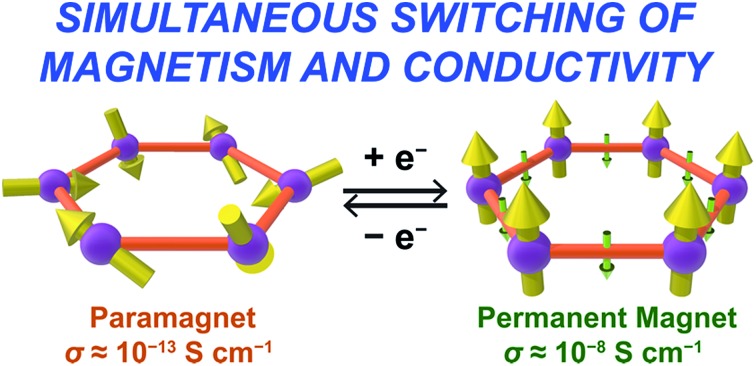
We report a 2D manganese benzoquinoid network that undergoes simultaneous redox switching of magnetic order and electrical conductivity.

## Introduction

Materials with switchable magnetic order or values of electrical conductivity underpin the realization of modern electronic and spintronic technologies.^1^ Within this class, materials that undergo simultaneous switching of both magnetism and conductivity are particularly attractive and have been the focus of active research in chemistry, condensed matter physics, and materials science.^2^ Indeed, such multi-switchable materials may lead to new technologies that are faster, more compact, and/or more energy efficient,^3^ such as terahertz information processing, magnetic transistors, and multifunctional chips where data storage and information processing can occur at the same location.^4^

Despite this potential, the realization of inorganic materials that possess switchable magnetic order associated with large changes in electrical conductivity faces several key challenges.^5^ For example, while the spin orientation of metals can be controlled using an external electric or magnetic field,^6^ their conductivity cannot be modulated due to the absence of a bandgap. In contrast, coercivities and magnetic ordering temperatures of dilute magnetic semiconductors or oxides can be controlled by an electric field.^7^ However, effective modulation of conductivity over several orders of magnitude in these materials is precluded by the presence of high charge carrier concentration arising from large degrees of transition metal doping.^8^ Notably, these limitations are largely imposed by a lack of chemical control over materials properties *via* pre-synthetic design or post-synthetic modification.2*d*

**Fig. 1:**
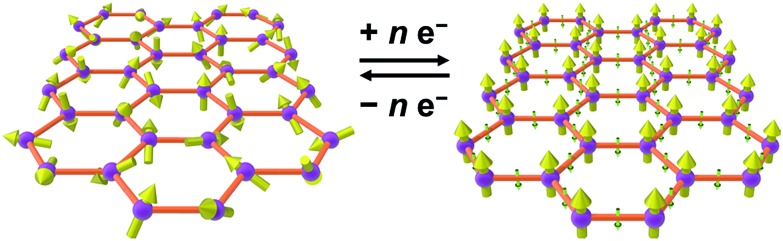
Graphical depiction of switching between paramagnetism (left) and magnetic order (right) through redox chemistry of organic linkers in a metal–organic framework.

As an alternative to inorganic compounds, metal–organic frameworks (MOFs) represent a powerful platform to design materials with tailored conductive and magnetic properties, owing to their extraordinary chemical versatility and tunability.^9^ In particular, the extensive palette of chemical space available in MOF synthesis offers the potential to incorporate redox-active metal nodes and/or organic linkers. Such incorporation of redox activity into the structure can give rise to redox-switchability, where properties are modulated by an electric field. For instance, one can envision a MOF that features paramagnetic metal centres and diamagnetic linkers and thus behaves as a paramagnet with low electrical conductivity, but can then undergo redox chemistry to also render the linkers paramagnetic. In this latter form, strong metal–radical coupling can lead to a permanent magnet with significantly increased conductivity by virtue of an increased number of charge carriers (see Fig. 1).

Although early examples of MOFs were nearly exclusively electrically insulating due to their composition of diamagnetic, redox-inert carboxylate linkers and metal nodes,^10^ recent years have seen significant progress in the discovery, understanding, and design of MOFs that exhibit electrical conductivity or magnetic order.^11^ For instance, reversible redox switching of magnetism has been demonstrated in (^*n*^Bu_4_N)[Cr^III^Mn^II^L_3_] (H_2_L = 2,5-dichloro-3,6-dihydroxo-1,4-benzoquinone)^12^ and in a Ru_2_ paddlewheel-TCNQ derivative.^13^ The former is a permanent magnet with an ordering temperature switchable from *T*_c_ = 10 to 33 K, whereas the latter undergoes switching between a paramagnet and a permanent magnet with *T*_c_ = 88 K. In addition, MOFs with high electrical conductivities have only very recently been realized.^14^ Within this class of conductive materials, redox switching of electrical conductivity has been reported,14b,^15^ and MOF-based field-effect transistors have been fabricated.14c,f,^16^

The coexistence of switchable magnetic order and electrical conductivity is exceedingly rare in any class of materials. For example, the magnetic ordering temperature of a dilute magnetic oxide Ti_0.9_Co_0.1_O_2_ can be controlled through electrolyte gating, where a maximum of 6-fold change in conductivity is observed.7*b* Recently, electrolyte gate-controlled ferromagnetism was reported in the 2D material Fe_3_GeTe_2_, where a similar up to 6-fold change in conductivity was observed in a 70-layer device.8*d* In addition, while gate-tuneable magnetism has been reported for CrI_3_ in several studies,^17^ its switchable electrical conductivity in this material has been only reported in one occasion.17*d* In two lone MOF examples, post-synthetic modification through the linker-based reduction of 3D^18^ and 2D^19^ ferric benzoquinoid magnets was shown to alter electrical conductivity by 26- and 27-fold, respectively. Nevertheless, magnetic order could not be switched off in these compounds, and methods to reverse the redox processes have not been reported. Here, we report just this type of redox switching in the framework compound (Me_4_N)_2_[Mn_2_L_3_], wherein reversible, post-synthetic chemical reduction transforms a paramagnet into a permanent magnet with *T*_c_ = 41 K, with a concomitant 200 000-fold enhancement of electrical conductivity. To our knowledge, this material is unique in its ability to simultaneously undergo redox switching of permanent magnetism and variation in electrical conductivity of several orders of magnitude.

## Results and discussion

### Synthesis and redox chemistry

**Fig. 2:**
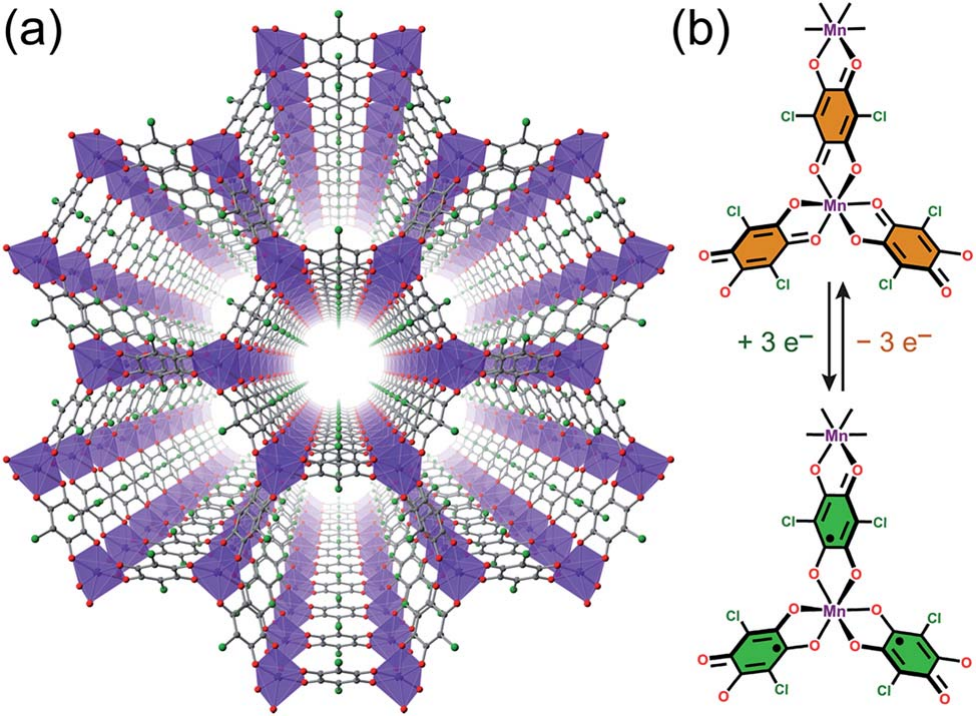
(a) Crystal structure of **1**, as viewed along the crystallographic *c* axis. Violet octahedra represent Mn atoms; green, red, and grey spheres represent Cl, O, and C atoms, respectively; cations and solvent molecules are omitted for clarity. (b) Molecular representation of quinoid-based redox chemistry described in this report, enabling switching between *S* = 0 and *S* = ½ linkers.

We selected the framework compound (Et_4_N)_2_[Mn_2_L_3_]·*x*DMF (**1**) as a platform to target switching of magnetism and conductivity, as it features exclusively *S* = 0 L^2–^ linkers between *S* = <svg xmlns="http://www.w3.org/2000/svg" version="1.0" width="16.000000pt" height="16.000000pt" viewBox="0 0 16.000000 16.000000" preserveAspectRatio="xMidYMid meet"><metadata> Created by potrace 1.16, written by Peter Selinger 2001-2019 </metadata><g transform="translate(1.000000,15.000000) scale(0.005147,-0.005147)" fill="currentColor" stroke="none"><path d="M240 2680 l0 -40 -40 0 -40 0 0 -280 0 -280 320 0 320 0 0 -40 0 -40 120 0 120 0 0 -40 0 -40 40 0 40 0 0 -80 0 -80 -80 0 -80 0 0 -80 0 -80 -240 0 -240 0 0 40 0 40 -80 0 -80 0 0 80 0 80 -80 0 -80 0 0 -80 0 -80 80 0 80 0 0 -40 0 -40 40 0 40 0 0 -40 0 -40 320 0 320 0 0 40 0 40 40 0 40 0 0 80 0 80 80 0 80 0 0 80 0 80 -40 0 -40 0 0 40 0 40 -40 0 -40 0 0 40 0 40 -120 0 -120 0 0 40 0 40 -280 0 -280 0 0 240 0 240 440 0 440 0 0 40 0 40 -480 0 -480 0 0 -40z M2640 2680 l0 -40 -40 0 -40 0 0 -40 0 -40 -40 0 -40 0 0 -40 0 -40 -40 0 -40 0 0 -40 0 -40 -40 0 -40 0 0 -40 0 -40 -40 0 -40 0 0 -40 0 -40 -40 0 -40 0 0 -40 0 -40 -40 0 -40 0 0 -40 0 -40 -40 0 -40 0 0 -40 0 -40 -40 0 -40 0 0 -40 0 -40 -40 0 -40 0 0 -40 0 -40 -40 0 -40 0 0 -40 0 -40 -40 0 -40 0 0 -40 0 -40 -40 0 -40 0 0 -40 0 -40 -40 0 -40 0 0 -40 0 -40 -40 0 -40 0 0 -40 0 -40 -40 0 -40 0 0 -40 0 -40 -40 0 -40 0 0 -40 0 -40 -40 0 -40 0 0 -40 0 -40 -40 0 -40 0 0 -40 0 -40 -40 0 -40 0 0 -40 0 -40 -40 0 -40 0 0 -40 0 -40 -40 0 -40 0 0 -40 0 -40 -40 0 -40 0 0 -40 0 -40 -40 0 -40 0 0 -40 0 -40 -40 0 -40 0 0 -40 0 -40 -40 0 -40 0 0 -40 0 -40 -40 0 -40 0 0 -40 0 -40 -40 0 -40 0 0 -40 0 -40 -40 0 -40 0 0 -40 0 -40 -40 0 -40 0 0 -40 0 -40 -40 0 -40 0 0 -40 0 -40 -40 0 -40 0 0 -40 0 -40 -40 0 -40 0 0 -40 0 -40 80 0 80 0 0 40 0 40 40 0 40 0 0 40 0 40 40 0 40 0 0 40 0 40 40 0 40 0 0 40 0 40 40 0 40 0 0 40 0 40 40 0 40 0 0 40 0 40 40 0 40 0 0 40 0 40 40 0 40 0 0 40 0 40 40 0 40 0 0 40 0 40 40 0 40 0 0 40 0 40 40 0 40 0 0 40 0 40 40 0 40 0 0 40 0 40 40 0 40 0 0 40 0 40 40 0 40 0 0 40 0 40 40 0 40 0 0 40 0 40 40 0 40 0 0 40 0 40 40 0 40 0 0 40 0 40 40 0 40 0 0 40 0 40 40 0 40 0 0 40 0 40 40 0 40 0 0 40 0 40 40 0 40 0 0 40 0 40 40 0 40 0 0 40 0 40 40 0 40 0 0 40 0 40 40 0 40 0 0 40 0 40 40 0 40 0 0 40 0 40 40 0 40 0 0 40 0 40 40 0 40 0 0 40 0 40 40 0 40 0 0 40 0 40 40 0 40 0 0 40 0 40 40 0 40 0 0 40 0 40 40 0 40 0 0 40 0 40 40 0 40 0 0 40 0 40 40 0 40 0 0 80 0 80 -40 0 -40 0 0 -40z M2080 1240 l0 -40 -80 0 -80 0 0 -40 0 -40 -80 0 -80 0 0 -80 0 -80 80 0 80 0 0 80 0 80 80 0 80 0 0 40 0 40 160 0 160 0 0 -40 0 -40 80 0 80 0 0 -200 0 -200 -80 0 -80 0 0 -40 0 -40 -80 0 -80 0 0 -40 0 -40 -80 0 -80 0 0 -40 0 -40 -80 0 -80 0 0 -40 0 -40 -40 0 -40 0 0 -40 0 -40 -40 0 -40 0 0 -160 0 -160 480 0 480 0 0 40 0 40 -400 0 -400 0 0 120 0 120 40 0 40 0 0 40 0 40 40 0 40 0 0 40 0 40 80 0 80 0 0 40 0 40 80 0 80 0 0 40 0 40 80 0 80 0 0 40 0 40 80 0 80 0 0 200 0 200 -80 0 -80 0 0 40 0 40 -80 0 -80 0 0 40 0 40 -160 0 -160 0 0 -40z"/></g></svg> Mn^II^ centres and should not exhibit magnetic order at finite temperatures owing to weak metal–metal interactions (see Fig. 2a).^20^ Yet, these linkers can potentially undergo reduction to their radical form to engender strong metal–radical coupling and thus magnetic order (see Fig. 2b).

**Fig. 3:**
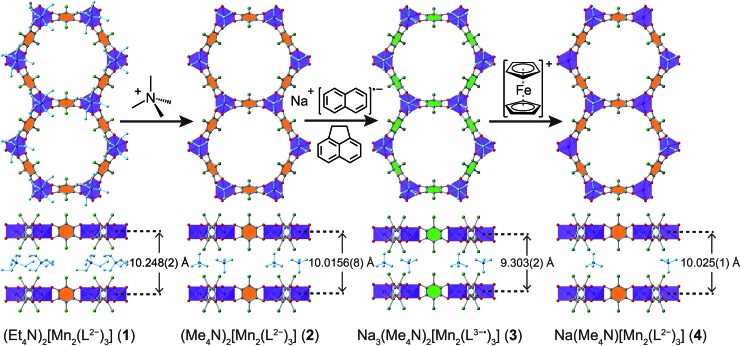
Crystal structures of **1–4** and the SC–SC conversions between them. Violet octahedra represent Mn atoms; green, red, and grey spheres represent Cl, O, and C atoms, respectively; Na^+^ ions that were not located in the crystal structures of **3** and **4** are not shown, and solvent molecules are omitted for clarity. Only one orientation of each disordered tetraalkylammonium ion is shown. Linkers with orange- and green-filled C6 rings represent L^2–^ and L^3–^˙, respectively.

Compound **1** was synthesized following a reported procedure.20*b* Attempts to reduce the linkers in **1** invariably led to incomplete reduction, likely due to the kinetic limitations imposed by the bulkiness of interstitial Et_4_N^+^ counterions. We therefore sought to synthesize an analogous framework with a smaller counterion. Toward this end, soaking crystals of **1** in a *N*,*N*-dimethylformamide (DMF) solution of (Me_4_N)BF_4_ at 75 °C for 40 h, followed by subsequent rinses with DMF and diethyl ether (Et_2_O), produced (Me_4_N)_2_[Mn_2_L_3_]·3.2Et_2_O (**2**) suitable for single-crystal X-ray diffraction analysis (see Fig. 3 and discussion below). The phase purity of **2** was confirmed by powder X-ray diffraction (PXRD, see Fig. S1†), while combustion elemental analysis and NMR spectroscopy confirmed the complete exchange of Et_4_N^+^ for Me_4_N^+^ (see ESI, Fig. S2 and S3†). Compound **2** could not be obtained in our hands through direct synthesis. When adapting the established synthetic protocol for compound **1** by replacing Et_4_N^+^ source with the corresponding Me_4_N^+^ salt, we obtained another crystalline compound. Hence, compound **2** was accessed through post-synthetic counterion exchange.

Chemical reduction of **2** was carried out by soaking crystals in an equimolar mixture of sodium naphthalenide and 1,2-dihydroacenaphthylene in THF at –35 °C for 23 days, which produced a colour change in the crystals from brown to dark green. Note that this combination of reductants was necessary to reach quantitative reduction. Although the exact identity of the active reductant is unclear, the reduction potentials of –3.23 V and –3.10 V *vs.* [Cp_2_Fe]^+/0^ for 1,2-dihydroacenaphthylene (see Fig. S4†) and naphthalene in THF,^21^ respectively, suggest that both C_10_H_8_˙^–^ and C_12_H_10_˙^–^ are present in the reaction with an estimated distribution of 12.6 : 1.

To determine the chemical formula and to tentatively assign the oxidation states of Mn and organic linker in the reduced compound, we employed inductively coupled plasma optical emission spectroscopy (ICP-OES), NMR spectroscopy, and combustion elemental analysis. First, ICP-OES analysis gave a Na : Mn ratio of 2.99 : 2, consistent with a three-electron reduction per Mn_3_L_2_ unit. In addition, NMR spectroscopy on a digested sample in DCl/DMSO-*d*_6_ identified the presence of Me_4_N^+^ and THF (see Fig. S5 and S6†), and importantly, the absence of DMF. Finally, combustion elemental analysis gave an overall formula of Na_3_(Me_4_N)_2_[Mn_2_L_3_]·3.9THF (**3**). Taken together, these data establish the composition of the 2D framework in **3** to be [Mn_2_L_3_]^5–^. Considering the Mn–O distance in **3** (see below) and the rare presence of Mn^I^ in molecular compounds^22^ and MOFs,^23^ we conclude that the oxidation state of Mn^II^ remains unchanged upon reduction. Instead, each L^2–^ undergoes a one-electron reduction to give the semiquinoid radical L^3–^˙, as is supported by the Raman spectroscopy and magnetometry described in detail below.

Soaking crystals of **3** in a solution of 3.3 equivalents of [Cp_2_Fe](BF_4_) in THF/CH_3_CN at ambient temperature for 6 days afforded brown hexagonal plate-shaped crystals of the re-oxidized material Na(Me_4_N)[Mn_2_L_3_]]∙5.5THF∙0.8CH5.5THF]∙5.5THF∙0.8CH0.8CH_3_CN (**4**). The Na : Mn ratio in **4** was determined to be 1.03 : 2 by ICP-OES, indicating removal of only 2/3 of the original Na^+^ counterions. From a charge-balance standpoint, a Me_4_N^+^ counterion is expected to remain in the compound, and its presence in **4** was confirmed by NMR spectroscopy (see Fig. S7 and S8†). The mixture of counterions in **4** suggests that half of the Me_4_N^+^ leached out of the crystals upon oxidation of **3**. To confirm this suggestion, the solvent of the reaction supernatant was removed under reduced pressure, and the presence of Me_4_N^+^ in the residual solid was confirmed by ^1^H and ^13^C NMR spectroscopy (see Fig. S9 and S10†), along with single-crystal X-ray diffraction (see below). Finally, this assignment of counterions was corroborated by combustion elemental analysis (see ESI†).

The formation of **2**, **3**, and **4***via* a dissolution–recrystallization mechanism can be ruled out based on several observations. First, since H_2_L is an organic dye with a high extinction coefficient, minor dissolution of the ligand would lead to a visible darkening of the supernatant.20*b* The fact that the supernatant remained colourless to the eye during the counterion exchange process strongly suggests that this exchange occurs *via* a single-crystal-to-single-crystal (SC–SC) transformation mechanism. Second, according to optical microscopy images, shown as insets of Fig. S11–S13,† typical sizes for hexagonal crystals of **2–4** across the *ab* planes are approximately 100 μm, and this unchanged crystal size and morphology further supports a SC–SC mechanism. Third, the reduction from **2** to **3** was performed in THF. Again, a dissolution–recrystallization mechanism is unlikely due to the poor solubility of the Mn^2+^, Me_4_N^+^, and L^2–^. Finally, according to the ^13^C NMR spectrum of the reaction supernatant from **3** to **4** (see Fig. S10†), the absence of the ligand peaks unambiguously confirms a SC–SC conversion mechanism.

### Crystal structures

The structures of **1–4** were determined using single-crystal X-ray diffraction experiments. The structures of both **1** and **2** were solved in the space group *P*3[combining macron]1*m*,20*b* and feature nearly identical structures, except for the counterion identity. As shown in Fig. 3, **2** features eclipsed-stacked 2D hexagonal [Mn_2_L_3_]^2–^ framework layers that are charge balanced with interstitial Me_4_N^+^ counterions positioned at the centre of two nearest Mn atoms from adjacent layers. Each Mn atom is coordinated to three bridging ligands through O atoms to form a pseudo-octahedral geometry. The unchanged Mn–O distance of 2.156(3) Å relative to that of 2.158(3) Å for **1** indicates the retention of the high-spin Mn^II^ upon counterion exchange (see Table 1). The average C–C bond length of the bridging ligand of **2** also remains unchanged relative to that of **1**, indicating the unchanged oxidation state of L^2–^.

**Table 1:** Unit cell parameters (upper, Å) and selected interatomic distances (lower, Å) for compounds **1–4**

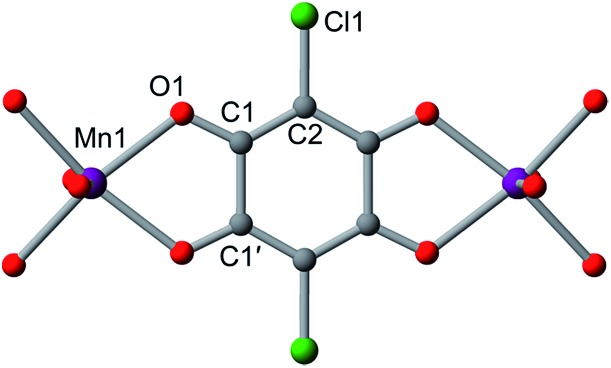				
	**1** (ref. 20*b*)	**2**	**3**	**4**
*a*	14.031(1)	14.034(1)	14.030(3)	14.083(1)
*c*	10.248(2)	10.0156(8)	9.303(2)	10.025(1)
Mn1–O1	2.158(3)	2.156(3)	2.15(2)	2.172(5)
O1–C1	1.254(7)	1.248(4)	1.29(3)	1.25(1)
C1–C2	1.376(6)	1.391(5)	1.33(4)	1.419(8)
C1–C1′	1.56(1)	1.538(7)	1.41(4)	1.52(1)
C–C_avg_	1.44(2)	1.44(1)	1.36(9)	1.45(2)

A single crystal of the reduced framework compound **3** diffracted moderately well, although the diffraction spots appeared more diffuse compared to those of **2** (see Fig. S11 and S12†), and with lower resolution. The structure of **3** was nevertheless solved in the same *P*3[combining macron]1*m* space group, with a shorter lattice parameter *c*, corresponding to an interlayer distance of 9.303(2) Å that is consistent with PXRD analysis (see below). The Me_4_N^+^ counterions occupy the same positions as in **2**. The Na^+^ ions could not be found in the difference electron density map, suggesting they are not ordered on any lattice positions. Bond length analyses further support the reduction of the ligand. As shown in Table 1, the C–O bond is slightly elongated, suggesting an increased single-bond character of the reduced ligand. All C–C bonds are shortened, especially C1–C1′, which contracts from 1.538(7) Å to 1.41(4) Å. The shortening of the C–C bonds is indicative of increased double bond character, also consistent the reduction of the L^2–^ to L^3–^˙.^24^

Remarkably, upon oxidation of **3** to **4**, the diffraction quality of **4** dramatically improved (see Fig. S13†), consistent with PXRD patterns of **3** and **4** (see below). The Me_4_N^+^ ion is situated at the same position as in **2** and **3**, but with 50% crystallographic occupancy. Similar to **3**, the remaining Na^+^ was not located in the electron density map, suggesting it is disordered within the pore space. While there is no contraction of the Mn–O bond, the C–O and C–C bonds are shortened and lengthened, respectively (see Table 1). These bond lengths are nearly identical to those in **2**, confirming the assignment of a ligand-based oxidation from L^3–^˙ to L^2–^.

### Powder X-ray diffraction

**Fig. 4:**
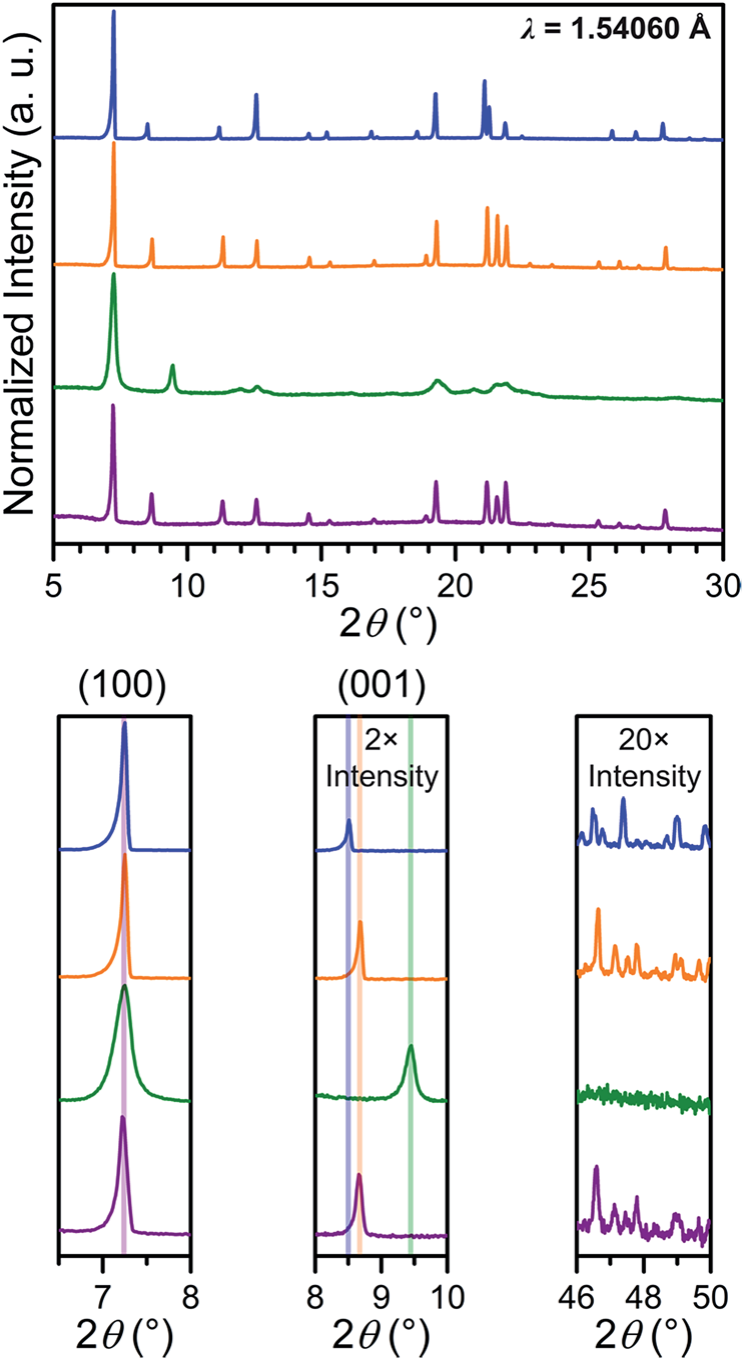
Power X-ray diffraction patterns of **1** (blue), **2** (orange), **3** (green) and **4** (purple), with expanded views of selected angle ranges shown below.

In order to probe the phase purity of crystalline **1–4** and to further investigate structural changes associated with counterion exchange and redox chemistry, these materials were examined using PXRD analysis (see Fig. 4). The peak at 7.250°, which corresponds to the (100) reflection, does not shift upon moving from **1** to **4**, indicating that lattice parameters *a* and *b* are unchanged through these reactions. Upon moving from **1** to **2**, the (001) peak shifts slightly toward higher angle, from 8.510° to 8.675°. This shift indicates a minor shortening of the interlayer distance by 0.20(3) Å, likely due to the smaller size of the Me_4_N^+^ in **2** compared to Et_4_N^+^ in **1**. The reduction from **2** to **3** results in an even more pronounced shift of the (001) peak, corresponding to a shortening from 10.18(2) to 9.35(1) Å. This interlayer contraction likely stems from increased electrostatic interactions between [Mn_2_L_3_]^5–^ layers and five interlayer cations, compared to two cations with [Mn_2_L_3_]^2–^ in **2**. As expected, the oxidation from **3** to **4** alters the charge of the framework from [Mn_2_L_3_]^5–^ to [Mn_2_L_3_]^2–^, and therefore causes an increase of the interlayer distance from 9.35(1) to 10.20(2) Å, identical to that of **2**.

The chemical reduction induces a significant peak broadening in **3**, which is most prominent in the range 2*θ* = 19–23°. Furthermore, in stark contrast to **2**, in which the high-angle diffraction peaks are well preserved upon counterion exchange, the high-angle peaks in **3** are completely extinguished upon reduction. For **3**, the increase in peak full width at half the maximum intensity (FWHM) at higher angle appears to be more pronounced than what is described by the Scherrer equation for crystallite-size related broadening, where FWHM is expected to be inversely proportional to cos *θ*. Instead, such a significant increase in FWHM seems proportional to tan *θ* (see Fig. S14†). From this tentative peak width analysis, we surmise that the peak broadening is more likely caused by the strain in **3**, rather than the reduction of the crystallite size. This suggestion is also evidenced from the optical microscopy images of **2** and **3** showing no change in size or morphology (see Fig. S11 and S12†). The significant increase in the strain of **3** is attributed to the accommodation of three additional counterions within the smaller lattice volume (see Table S1†). This likely causes misalignment of individual lattices within each crystal, giving rise to more mosaic behaviour, which is also supported by the more diffused single-crystal diffraction patterns (see Fig. S12†).

Upon oxidation, these high-angle peaks surprisingly reappear for **4** at identical angles as in **2**. Moreover, the peaks in **4** are significantly sharper than **3** throughout entire 2*θ* range. Thus, the oxidation from **3** to **4** can restore apparent crystallinity. This can be explained by the expulsion of three counterions and concomitant increase in unit cell volume. Consequently, the steric conflict caused by the strong electrostatic interactions and steric conflicts in **3** is significantly relieved in **4** to relax the strain.

### Raman spectroscopy

**Fig. 5:**
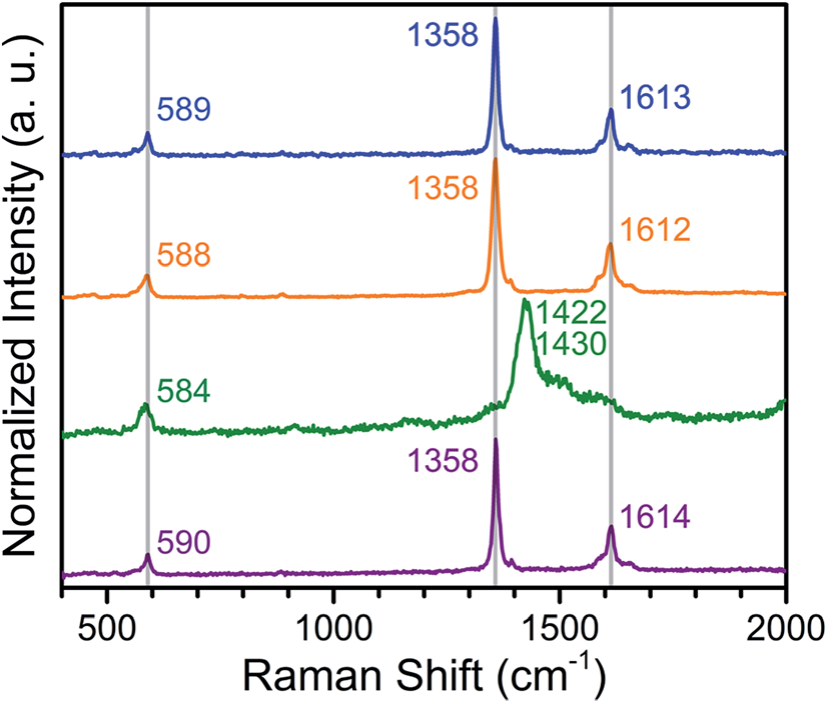
Raman spectra collected for solid samples of **1** (blue), **2** (orange), **3** (green), and **4** (purple), following excitation at a wavelength of 473 nm. Grey lines are guides to the eye.

To confirm the ligand oxidation state assignments for **1–4**, we collected solid-state Raman spectra for the compounds, as the Raman shifts associated with the C–C and C–O vibrations can provide diagnostic information regarding the oxidation state of quinoid ligands in both molecular complexes and MOFs.^19^,^25^ The Raman bands centred at 1358 and 1612–1614 cm^–1^ are assigned to be the C–C and C–O stretch, respectively, in **1**, **2**, and **4** (see Fig. 5). Based on similar corresponding Raman bands at 1360 and 1617 cm^–1^ for (Me_2_NH_2_)_2_[ZnII2(L^2–^)_3_],19*a* the ligand oxidation states of L^2–^ in **1**, **2**, and **4** are unambiguously confirmed. Upon reduction, the C–C bonds and the C–O bond are contracted and elongated, respectively, in **3**, as determined above from X-ray diffraction. Consequently, the corresponding vibrations should shift toward higher and lower energy, respectively. Only one prominent band is present in the spectrum of **3**, centred at 1426 cm^–1^. This band most likely corresponds to overlapping peaks for both C–C and C–O stretches of L^3–^˙. Although these vibrations in **3** are slightly shifted relative to those of *ν*_C–C_ = 1390 and *ν*_C–O_ = 1488 cm^–1^ observed for (Cp_2_Co^III^)_1.43_(Me_2_NH_2_)_1.57_[FeIII2(L^3–^˙)_3_],19*b* the presence of a similar prominent band was observed at 1426 cm^–1^ for the dinuclear complex [CrIII2(tren)_2_(L^3–^˙)]^3+^ (tren = tris(2-aminoethyl)amine).25*b* Taken together, the foregoing experiments unambiguously confirm the oxidation state assignment of the ligand as L^2–^ in **1**, **2**, and **4**, and as L^3–^˙ in **3**.

### Electrical conductivity

**Fig. 6:**
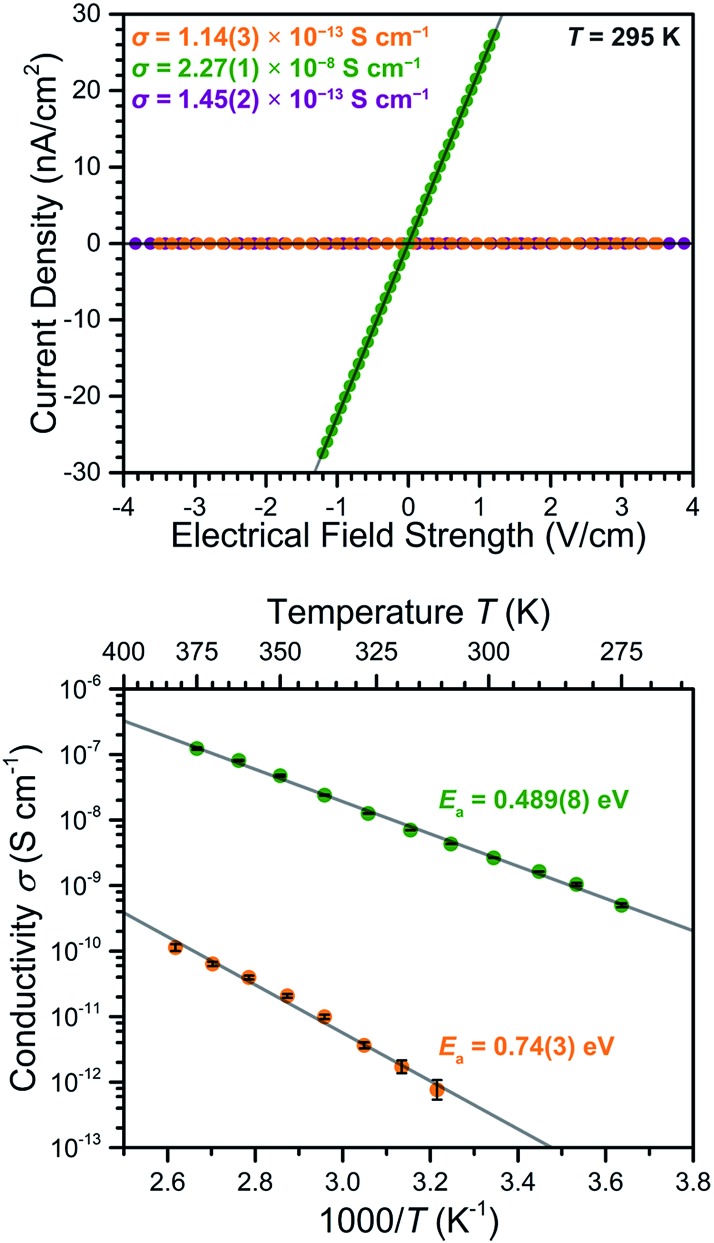
Upper: Two-point pressed-pellet current-bias plot measured at 295 K for **2** (orange), **3** (green), and **4** (purple). Lower: Variable-temperature conductivity data obtained for **2** (orange) and **3** (green). Grey lines represent fits to the data.

To probe the impact of the ligand oxidation state on the electronic communication and transport in these frameworks, two-contact-probe conductivity measurements were carried out for pressed pellets of **2–4** at 295 K (see Fig. 6, upper). For **2** and **4**, which feature exclusively diamagnetic L^2–^ linkers, the conductivities were measured to be *σ*_295 K_ = 1.14(3) × 10^–13^ and 1.45(2) × 10^–13^ S cm^–1^, respectively. The nearly identical conductivity values for **2** and **4** support the conclusion that the redox switchability of conductivity is close to completely reversible. These low conductivity values fall in the typical range observed for Mn^II^-based MOFs with diamagnetic linkers.^26^

The reduced compound **3** exhibited a conductivity of *σ*_295 K_ = 2.27(1) × 10^–8^ S cm^–1^, representing a 200 000-fold increase relative to the parent compound **2**. To our knowledge, this is the largest change in electrical conductivity stemming from ligand-based redox chemistry in a MOF. This large increase upon chemical reduction indicates that reduction effectively injects electrons into the framework and thereby dramatically improves its charge carrier density. Notably, the ambient temperature conductivity value of **3** is among the highest of structurally-characterized Mn-based MOFs, and is only eclipsed by the value of *σ* = 8(1) × 10^–5^ S cm^–1^ reported for a single crystal of Mn_2_(TTFTB) (TTFTB = tetrathiafulvalene tetrabenzoate). Note that the observed pressed-pellet conductivity values for low-dimensional materials, such as **3**, are typically several orders of magnitude lower than single-crystal measurements, due to the anisotropic conducting pathway and grain boundaries.11g,^27^ Accordingly, the conductivity across the 2D framework in **3** is likely much higher than the value for the pellet.

The conductivity value of **3** is comparable to the 1D radical-bridged chain compound (Cp_2_Co)Mn(^N,O^L) [H_4_^N,O^L = 4,5-bis(pyridine-2-carboxamido)-1,2-catechol], which exhibits an ambient-temperature conductivity of *σ* = 8.6 × 10^–8^ S cm^–1^.^28^ In contrast, the conductivity of **3** is 2.2 × 10^4^ times lower than that of the related compound (Cp_2_Co)_1.43_(Me_2_NH_2_)_1.57_[FeIII2(L^3–^˙)_3_], which exhibits a pressed-pellet conductivity of *σ*_300 K_ = 5.1(3) × 10^–4^ S cm^–1^.19*b* This difference is likely due in large part to the expected closer redox potential between Fe^3+^/Fe^2+^ and L^2–^/L^3–^˙ pairs relative to Mn^3+^/Mn^2+^ and L^2–^/L^3–^˙ pairs. In particular, the better energetic match of redox potential in the former pair might allow ligand radical electrons to transport through the Fe^3+^ centres *via* a transient valence tautomerism mechanism,^29^ whereas such a transport pathway is narrower in the Mn frameworks described here.

To further probe the origin of the electrical conductivity, variable-temperature two-contact-probe pressed-pellet conductivities were measured in the temperature range of 310–385 K for **2**, and 275–375 K for **3**, respectively. As depicted in the lower panel of Fig. 6, linear relationships between the logarithm of conductivity and inverse temperature are observed for both **2** and **3**, suggesting the presence of thermally-activated charge transport in both materials. Fitting the data using the Arrhenius equation (see ESI† for details) gave activation energies of *E*_a_ = 0.74(3) eV for **2** and 0.489(8) eV for **3**, respectively. The lowering of the activation energy upon reduction is likely due to the Fermi level rising closer to the conduction band as a consequence of doping electrons into the material.^30^

The 200 000-fold redox switching of electrical conductivity in (Me_4_N)_2_[Mn_2_L_3_] is remarkable among magnets. As noted above, the ability to modulate electrical conductivity values over several orders of magnitude in permanent magnets constitutes a significant synthetic challenge, as low values of on/off ratio typically observed for inorganic compounds have hampered their practical use in field-effect transistors. For example, a six-fold change in conductivity was observed at 300 K for the dilute magnetic oxide Ti_0.9_Co_0.1_O_2_ upon applying a gate voltage of 3.8 V through an ionic liquid,7*b* while a similar enhancement in conductivity was observed in 70-layer Fe_3_GeTe_2_ upon applying a 5 V bias through an ionic gate.8*d*

### Magnetic properties

To probe the magnetic behaviour of the manganese-quinoid compounds, variable-temperature dc magnetic susceptibility data were collected for **2–4**. As shown in Fig. S15,† the *χ*_M_*T* values for **2** and **4** at 300 K are 8.74 and 8.69 cm^3^ mol^–1^ K respectively, very close to the expected value of 8.75 cm^3^ mol^–1^ K for two isolated *S* = <svg xmlns="http://www.w3.org/2000/svg" version="1.0" width="16.000000pt" height="16.000000pt" viewBox="0 0 16.000000 16.000000" preserveAspectRatio="xMidYMid meet"><metadata> Created by potrace 1.16, written by Peter Selinger 2001-2019 </metadata><g transform="translate(1.000000,15.000000) scale(0.005147,-0.005147)" fill="currentColor" stroke="none"><path d="M240 2680 l0 -40 -40 0 -40 0 0 -280 0 -280 320 0 320 0 0 -40 0 -40 120 0 120 0 0 -40 0 -40 40 0 40 0 0 -80 0 -80 -80 0 -80 0 0 -80 0 -80 -240 0 -240 0 0 40 0 40 -80 0 -80 0 0 80 0 80 -80 0 -80 0 0 -80 0 -80 80 0 80 0 0 -40 0 -40 40 0 40 0 0 -40 0 -40 320 0 320 0 0 40 0 40 40 0 40 0 0 80 0 80 80 0 80 0 0 80 0 80 -40 0 -40 0 0 40 0 40 -40 0 -40 0 0 40 0 40 -120 0 -120 0 0 40 0 40 -280 0 -280 0 0 240 0 240 440 0 440 0 0 40 0 40 -480 0 -480 0 0 -40z M2640 2680 l0 -40 -40 0 -40 0 0 -40 0 -40 -40 0 -40 0 0 -40 0 -40 -40 0 -40 0 0 -40 0 -40 -40 0 -40 0 0 -40 0 -40 -40 0 -40 0 0 -40 0 -40 -40 0 -40 0 0 -40 0 -40 -40 0 -40 0 0 -40 0 -40 -40 0 -40 0 0 -40 0 -40 -40 0 -40 0 0 -40 0 -40 -40 0 -40 0 0 -40 0 -40 -40 0 -40 0 0 -40 0 -40 -40 0 -40 0 0 -40 0 -40 -40 0 -40 0 0 -40 0 -40 -40 0 -40 0 0 -40 0 -40 -40 0 -40 0 0 -40 0 -40 -40 0 -40 0 0 -40 0 -40 -40 0 -40 0 0 -40 0 -40 -40 0 -40 0 0 -40 0 -40 -40 0 -40 0 0 -40 0 -40 -40 0 -40 0 0 -40 0 -40 -40 0 -40 0 0 -40 0 -40 -40 0 -40 0 0 -40 0 -40 -40 0 -40 0 0 -40 0 -40 -40 0 -40 0 0 -40 0 -40 -40 0 -40 0 0 -40 0 -40 -40 0 -40 0 0 -40 0 -40 -40 0 -40 0 0 -40 0 -40 -40 0 -40 0 0 -40 0 -40 -40 0 -40 0 0 -40 0 -40 -40 0 -40 0 0 -40 0 -40 -40 0 -40 0 0 -40 0 -40 -40 0 -40 0 0 -40 0 -40 -40 0 -40 0 0 -40 0 -40 80 0 80 0 0 40 0 40 40 0 40 0 0 40 0 40 40 0 40 0 0 40 0 40 40 0 40 0 0 40 0 40 40 0 40 0 0 40 0 40 40 0 40 0 0 40 0 40 40 0 40 0 0 40 0 40 40 0 40 0 0 40 0 40 40 0 40 0 0 40 0 40 40 0 40 0 0 40 0 40 40 0 40 0 0 40 0 40 40 0 40 0 0 40 0 40 40 0 40 0 0 40 0 40 40 0 40 0 0 40 0 40 40 0 40 0 0 40 0 40 40 0 40 0 0 40 0 40 40 0 40 0 0 40 0 40 40 0 40 0 0 40 0 40 40 0 40 0 0 40 0 40 40 0 40 0 0 40 0 40 40 0 40 0 0 40 0 40 40 0 40 0 0 40 0 40 40 0 40 0 0 40 0 40 40 0 40 0 0 40 0 40 40 0 40 0 0 40 0 40 40 0 40 0 0 40 0 40 40 0 40 0 0 40 0 40 40 0 40 0 0 40 0 40 40 0 40 0 0 40 0 40 40 0 40 0 0 40 0 40 40 0 40 0 0 40 0 40 40 0 40 0 0 40 0 40 40 0 40 0 0 80 0 80 -40 0 -40 0 0 -40z M2080 1240 l0 -40 -80 0 -80 0 0 -40 0 -40 -80 0 -80 0 0 -80 0 -80 80 0 80 0 0 80 0 80 80 0 80 0 0 40 0 40 160 0 160 0 0 -40 0 -40 80 0 80 0 0 -200 0 -200 -80 0 -80 0 0 -40 0 -40 -80 0 -80 0 0 -40 0 -40 -80 0 -80 0 0 -40 0 -40 -80 0 -80 0 0 -40 0 -40 -40 0 -40 0 0 -40 0 -40 -40 0 -40 0 0 -160 0 -160 480 0 480 0 0 40 0 40 -400 0 -400 0 0 120 0 120 40 0 40 0 0 40 0 40 40 0 40 0 0 40 0 40 80 0 80 0 0 40 0 40 80 0 80 0 0 40 0 40 80 0 80 0 0 40 0 40 80 0 80 0 0 200 0 200 -80 0 -80 0 0 40 0 40 -80 0 -80 0 0 40 0 40 -160 0 -160 0 0 -40z"/></g></svg> Mn^II^ ions per formula unit with *g* = 2. Upon lowering the temperature, *χ*_M_*T* for **2** and **4** remains nearly constant down to *ca.* 150 K. Below 150 K, *χ*_M_*T* for **2** undergoes a monotonic decrease to reach a minimum value of *χ*_M_*T* = 0.74 cm^3^ mol^–1^ K at 2 K, indicating relatively weak antiferromagnetic superexchange coupling between *S* = <svg xmlns="http://www.w3.org/2000/svg" version="1.0" width="16.000000pt" height="16.000000pt" viewBox="0 0 16.000000 16.000000" preserveAspectRatio="xMidYMid meet"><metadata> Created by potrace 1.16, written by Peter Selinger 2001-2019 </metadata><g transform="translate(1.000000,15.000000) scale(0.005147,-0.005147)" fill="currentColor" stroke="none"><path d="M240 2680 l0 -40 -40 0 -40 0 0 -280 0 -280 320 0 320 0 0 -40 0 -40 120 0 120 0 0 -40 0 -40 40 0 40 0 0 -80 0 -80 -80 0 -80 0 0 -80 0 -80 -240 0 -240 0 0 40 0 40 -80 0 -80 0 0 80 0 80 -80 0 -80 0 0 -80 0 -80 80 0 80 0 0 -40 0 -40 40 0 40 0 0 -40 0 -40 320 0 320 0 0 40 0 40 40 0 40 0 0 80 0 80 80 0 80 0 0 80 0 80 -40 0 -40 0 0 40 0 40 -40 0 -40 0 0 40 0 40 -120 0 -120 0 0 40 0 40 -280 0 -280 0 0 240 0 240 440 0 440 0 0 40 0 40 -480 0 -480 0 0 -40z M2640 2680 l0 -40 -40 0 -40 0 0 -40 0 -40 -40 0 -40 0 0 -40 0 -40 -40 0 -40 0 0 -40 0 -40 -40 0 -40 0 0 -40 0 -40 -40 0 -40 0 0 -40 0 -40 -40 0 -40 0 0 -40 0 -40 -40 0 -40 0 0 -40 0 -40 -40 0 -40 0 0 -40 0 -40 -40 0 -40 0 0 -40 0 -40 -40 0 -40 0 0 -40 0 -40 -40 0 -40 0 0 -40 0 -40 -40 0 -40 0 0 -40 0 -40 -40 0 -40 0 0 -40 0 -40 -40 0 -40 0 0 -40 0 -40 -40 0 -40 0 0 -40 0 -40 -40 0 -40 0 0 -40 0 -40 -40 0 -40 0 0 -40 0 -40 -40 0 -40 0 0 -40 0 -40 -40 0 -40 0 0 -40 0 -40 -40 0 -40 0 0 -40 0 -40 -40 0 -40 0 0 -40 0 -40 -40 0 -40 0 0 -40 0 -40 -40 0 -40 0 0 -40 0 -40 -40 0 -40 0 0 -40 0 -40 -40 0 -40 0 0 -40 0 -40 -40 0 -40 0 0 -40 0 -40 -40 0 -40 0 0 -40 0 -40 -40 0 -40 0 0 -40 0 -40 -40 0 -40 0 0 -40 0 -40 -40 0 -40 0 0 -40 0 -40 -40 0 -40 0 0 -40 0 -40 -40 0 -40 0 0 -40 0 -40 -40 0 -40 0 0 -40 0 -40 80 0 80 0 0 40 0 40 40 0 40 0 0 40 0 40 40 0 40 0 0 40 0 40 40 0 40 0 0 40 0 40 40 0 40 0 0 40 0 40 40 0 40 0 0 40 0 40 40 0 40 0 0 40 0 40 40 0 40 0 0 40 0 40 40 0 40 0 0 40 0 40 40 0 40 0 0 40 0 40 40 0 40 0 0 40 0 40 40 0 40 0 0 40 0 40 40 0 40 0 0 40 0 40 40 0 40 0 0 40 0 40 40 0 40 0 0 40 0 40 40 0 40 0 0 40 0 40 40 0 40 0 0 40 0 40 40 0 40 0 0 40 0 40 40 0 40 0 0 40 0 40 40 0 40 0 0 40 0 40 40 0 40 0 0 40 0 40 40 0 40 0 0 40 0 40 40 0 40 0 0 40 0 40 40 0 40 0 0 40 0 40 40 0 40 0 0 40 0 40 40 0 40 0 0 40 0 40 40 0 40 0 0 40 0 40 40 0 40 0 0 40 0 40 40 0 40 0 0 40 0 40 40 0 40 0 0 40 0 40 40 0 40 0 0 40 0 40 40 0 40 0 0 40 0 40 40 0 40 0 0 80 0 80 -40 0 -40 0 0 -40z M2080 1240 l0 -40 -80 0 -80 0 0 -40 0 -40 -80 0 -80 0 0 -80 0 -80 80 0 80 0 0 80 0 80 80 0 80 0 0 40 0 40 160 0 160 0 0 -40 0 -40 80 0 80 0 0 -200 0 -200 -80 0 -80 0 0 -40 0 -40 -80 0 -80 0 0 -40 0 -40 -80 0 -80 0 0 -40 0 -40 -80 0 -80 0 0 -40 0 -40 -40 0 -40 0 0 -40 0 -40 -40 0 -40 0 0 -160 0 -160 480 0 480 0 0 40 0 40 -400 0 -400 0 0 120 0 120 40 0 40 0 0 40 0 40 40 0 40 0 0 40 0 40 80 0 80 0 0 40 0 40 80 0 80 0 0 40 0 40 80 0 80 0 0 40 0 40 80 0 80 0 0 200 0 200 -80 0 -80 0 0 40 0 40 -80 0 -80 0 0 40 0 40 -160 0 -160 0 0 -40z"/></g></svg> Mn^II^ ions. As such, **2** is a paramagnet at temperatures down to at least 2 K. Conversely, in the case of **4**, *χ*_M_*T* undergoes a slight increase below 90 K to a maximum value of *χ*_M_*T* = 9.59 cm^3^ mol^–1^ K at 40 K, followed by a monotonic decrease to reach a minimum value of *χ*_M_*T* = 1.09 cm^3^ mol^–1^ K at 2 K. This slight upturn in **4** upon lowering the temperature is most likely due to the contribution from a minuscule amount of reduced species remaining in the sample (see below).

For **3**, the value of *χ*_M_*T* = 9.60 cm^3^ mol^–1^ K at 300 K is relatively close to that of 9.875 cm^3^ mol^–1^ K expected for magnetically isolated two *S* = <svg xmlns="http://www.w3.org/2000/svg" version="1.0" width="16.000000pt" height="16.000000pt" viewBox="0 0 16.000000 16.000000" preserveAspectRatio="xMidYMid meet"><metadata> Created by potrace 1.16, written by Peter Selinger 2001-2019 </metadata><g transform="translate(1.000000,15.000000) scale(0.005147,-0.005147)" fill="currentColor" stroke="none"><path d="M240 2680 l0 -40 -40 0 -40 0 0 -280 0 -280 320 0 320 0 0 -40 0 -40 120 0 120 0 0 -40 0 -40 40 0 40 0 0 -80 0 -80 -80 0 -80 0 0 -80 0 -80 -240 0 -240 0 0 40 0 40 -80 0 -80 0 0 80 0 80 -80 0 -80 0 0 -80 0 -80 80 0 80 0 0 -40 0 -40 40 0 40 0 0 -40 0 -40 320 0 320 0 0 40 0 40 40 0 40 0 0 80 0 80 80 0 80 0 0 80 0 80 -40 0 -40 0 0 40 0 40 -40 0 -40 0 0 40 0 40 -120 0 -120 0 0 40 0 40 -280 0 -280 0 0 240 0 240 440 0 440 0 0 40 0 40 -480 0 -480 0 0 -40z M2640 2680 l0 -40 -40 0 -40 0 0 -40 0 -40 -40 0 -40 0 0 -40 0 -40 -40 0 -40 0 0 -40 0 -40 -40 0 -40 0 0 -40 0 -40 -40 0 -40 0 0 -40 0 -40 -40 0 -40 0 0 -40 0 -40 -40 0 -40 0 0 -40 0 -40 -40 0 -40 0 0 -40 0 -40 -40 0 -40 0 0 -40 0 -40 -40 0 -40 0 0 -40 0 -40 -40 0 -40 0 0 -40 0 -40 -40 0 -40 0 0 -40 0 -40 -40 0 -40 0 0 -40 0 -40 -40 0 -40 0 0 -40 0 -40 -40 0 -40 0 0 -40 0 -40 -40 0 -40 0 0 -40 0 -40 -40 0 -40 0 0 -40 0 -40 -40 0 -40 0 0 -40 0 -40 -40 0 -40 0 0 -40 0 -40 -40 0 -40 0 0 -40 0 -40 -40 0 -40 0 0 -40 0 -40 -40 0 -40 0 0 -40 0 -40 -40 0 -40 0 0 -40 0 -40 -40 0 -40 0 0 -40 0 -40 -40 0 -40 0 0 -40 0 -40 -40 0 -40 0 0 -40 0 -40 -40 0 -40 0 0 -40 0 -40 -40 0 -40 0 0 -40 0 -40 -40 0 -40 0 0 -40 0 -40 -40 0 -40 0 0 -40 0 -40 -40 0 -40 0 0 -40 0 -40 -40 0 -40 0 0 -40 0 -40 -40 0 -40 0 0 -40 0 -40 80 0 80 0 0 40 0 40 40 0 40 0 0 40 0 40 40 0 40 0 0 40 0 40 40 0 40 0 0 40 0 40 40 0 40 0 0 40 0 40 40 0 40 0 0 40 0 40 40 0 40 0 0 40 0 40 40 0 40 0 0 40 0 40 40 0 40 0 0 40 0 40 40 0 40 0 0 40 0 40 40 0 40 0 0 40 0 40 40 0 40 0 0 40 0 40 40 0 40 0 0 40 0 40 40 0 40 0 0 40 0 40 40 0 40 0 0 40 0 40 40 0 40 0 0 40 0 40 40 0 40 0 0 40 0 40 40 0 40 0 0 40 0 40 40 0 40 0 0 40 0 40 40 0 40 0 0 40 0 40 40 0 40 0 0 40 0 40 40 0 40 0 0 40 0 40 40 0 40 0 0 40 0 40 40 0 40 0 0 40 0 40 40 0 40 0 0 40 0 40 40 0 40 0 0 40 0 40 40 0 40 0 0 40 0 40 40 0 40 0 0 40 0 40 40 0 40 0 0 40 0 40 40 0 40 0 0 40 0 40 40 0 40 0 0 40 0 40 40 0 40 0 0 40 0 40 40 0 40 0 0 80 0 80 -40 0 -40 0 0 -40z M2080 1240 l0 -40 -80 0 -80 0 0 -40 0 -40 -80 0 -80 0 0 -80 0 -80 80 0 80 0 0 80 0 80 80 0 80 0 0 40 0 40 160 0 160 0 0 -40 0 -40 80 0 80 0 0 -200 0 -200 -80 0 -80 0 0 -40 0 -40 -80 0 -80 0 0 -40 0 -40 -80 0 -80 0 0 -40 0 -40 -80 0 -80 0 0 -40 0 -40 -40 0 -40 0 0 -40 0 -40 -40 0 -40 0 0 -160 0 -160 480 0 480 0 0 40 0 40 -400 0 -400 0 0 120 0 120 40 0 40 0 0 40 0 40 40 0 40 0 0 40 0 40 80 0 80 0 0 40 0 40 80 0 80 0 0 40 0 40 80 0 80 0 0 40 0 40 80 0 80 0 0 200 0 200 -80 0 -80 0 0 40 0 40 -80 0 -80 0 0 40 0 40 -160 0 -160 0 0 -40z"/></g></svg> Mn^II^ ions and three S = ½ L^3–^˙ radicals per formula unit (see Fig. S16†). In stark contrast to **2** and **4**, the *χ*_M_*T* value for **3** increases monotonically upon lowering temperature to reach a maximum value of *χ*_M_*T* = 2570 cm^3^ mol^–1^ K at 26 K under an applied dc field of *H* = 10 Oe. This behaviour indicates the presence of long-range magnetic correlation between spins.

To confirm the quantitative ligand-based reduction and to further investigate the nature of magnetic coupling in **3**, variable-field magnetization data were measured at 1.8 K. The resulting *M vs. H* curve saturates at a value of *M* = 7.00 *μ*_B_ at 7 T (see Fig. S17†), which corresponds precisely to the value expected for a repeating unit containing two *S* = <svg xmlns="http://www.w3.org/2000/svg" version="1.0" width="16.000000pt" height="16.000000pt" viewBox="0 0 16.000000 16.000000" preserveAspectRatio="xMidYMid meet"><metadata> Created by potrace 1.16, written by Peter Selinger 2001-2019 </metadata><g transform="translate(1.000000,15.000000) scale(0.005147,-0.005147)" fill="currentColor" stroke="none"><path d="M240 2680 l0 -40 -40 0 -40 0 0 -280 0 -280 320 0 320 0 0 -40 0 -40 120 0 120 0 0 -40 0 -40 40 0 40 0 0 -80 0 -80 -80 0 -80 0 0 -80 0 -80 -240 0 -240 0 0 40 0 40 -80 0 -80 0 0 80 0 80 -80 0 -80 0 0 -80 0 -80 80 0 80 0 0 -40 0 -40 40 0 40 0 0 -40 0 -40 320 0 320 0 0 40 0 40 40 0 40 0 0 80 0 80 80 0 80 0 0 80 0 80 -40 0 -40 0 0 40 0 40 -40 0 -40 0 0 40 0 40 -120 0 -120 0 0 40 0 40 -280 0 -280 0 0 240 0 240 440 0 440 0 0 40 0 40 -480 0 -480 0 0 -40z M2640 2680 l0 -40 -40 0 -40 0 0 -40 0 -40 -40 0 -40 0 0 -40 0 -40 -40 0 -40 0 0 -40 0 -40 -40 0 -40 0 0 -40 0 -40 -40 0 -40 0 0 -40 0 -40 -40 0 -40 0 0 -40 0 -40 -40 0 -40 0 0 -40 0 -40 -40 0 -40 0 0 -40 0 -40 -40 0 -40 0 0 -40 0 -40 -40 0 -40 0 0 -40 0 -40 -40 0 -40 0 0 -40 0 -40 -40 0 -40 0 0 -40 0 -40 -40 0 -40 0 0 -40 0 -40 -40 0 -40 0 0 -40 0 -40 -40 0 -40 0 0 -40 0 -40 -40 0 -40 0 0 -40 0 -40 -40 0 -40 0 0 -40 0 -40 -40 0 -40 0 0 -40 0 -40 -40 0 -40 0 0 -40 0 -40 -40 0 -40 0 0 -40 0 -40 -40 0 -40 0 0 -40 0 -40 -40 0 -40 0 0 -40 0 -40 -40 0 -40 0 0 -40 0 -40 -40 0 -40 0 0 -40 0 -40 -40 0 -40 0 0 -40 0 -40 -40 0 -40 0 0 -40 0 -40 -40 0 -40 0 0 -40 0 -40 -40 0 -40 0 0 -40 0 -40 -40 0 -40 0 0 -40 0 -40 -40 0 -40 0 0 -40 0 -40 -40 0 -40 0 0 -40 0 -40 -40 0 -40 0 0 -40 0 -40 -40 0 -40 0 0 -40 0 -40 80 0 80 0 0 40 0 40 40 0 40 0 0 40 0 40 40 0 40 0 0 40 0 40 40 0 40 0 0 40 0 40 40 0 40 0 0 40 0 40 40 0 40 0 0 40 0 40 40 0 40 0 0 40 0 40 40 0 40 0 0 40 0 40 40 0 40 0 0 40 0 40 40 0 40 0 0 40 0 40 40 0 40 0 0 40 0 40 40 0 40 0 0 40 0 40 40 0 40 0 0 40 0 40 40 0 40 0 0 40 0 40 40 0 40 0 0 40 0 40 40 0 40 0 0 40 0 40 40 0 40 0 0 40 0 40 40 0 40 0 0 40 0 40 40 0 40 0 0 40 0 40 40 0 40 0 0 40 0 40 40 0 40 0 0 40 0 40 40 0 40 0 0 40 0 40 40 0 40 0 0 40 0 40 40 0 40 0 0 40 0 40 40 0 40 0 0 40 0 40 40 0 40 0 0 40 0 40 40 0 40 0 0 40 0 40 40 0 40 0 0 40 0 40 40 0 40 0 0 40 0 40 40 0 40 0 0 40 0 40 40 0 40 0 0 40 0 40 40 0 40 0 0 40 0 40 40 0 40 0 0 80 0 80 -40 0 -40 0 0 -40z M2080 1240 l0 -40 -80 0 -80 0 0 -40 0 -40 -80 0 -80 0 0 -80 0 -80 80 0 80 0 0 80 0 80 80 0 80 0 0 40 0 40 160 0 160 0 0 -40 0 -40 80 0 80 0 0 -200 0 -200 -80 0 -80 0 0 -40 0 -40 -80 0 -80 0 0 -40 0 -40 -80 0 -80 0 0 -40 0 -40 -80 0 -80 0 0 -40 0 -40 -40 0 -40 0 0 -40 0 -40 -40 0 -40 0 0 -160 0 -160 480 0 480 0 0 40 0 40 -400 0 -400 0 0 120 0 120 40 0 40 0 0 40 0 40 40 0 40 0 0 40 0 40 80 0 80 0 0 40 0 40 80 0 80 0 0 40 0 40 80 0 80 0 0 40 0 40 80 0 80 0 0 200 0 200 -80 0 -80 0 0 40 0 40 -80 0 -80 0 0 40 0 40 -160 0 -160 0 0 -40z"/></g></svg> Mn^II^ centres that are antiferromagnetically coupled to three S = ½ L^3–^˙ radical ligands, giving a net repeating spin of 
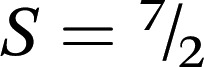
 and *g* = 2.00.

**Fig. 7:**
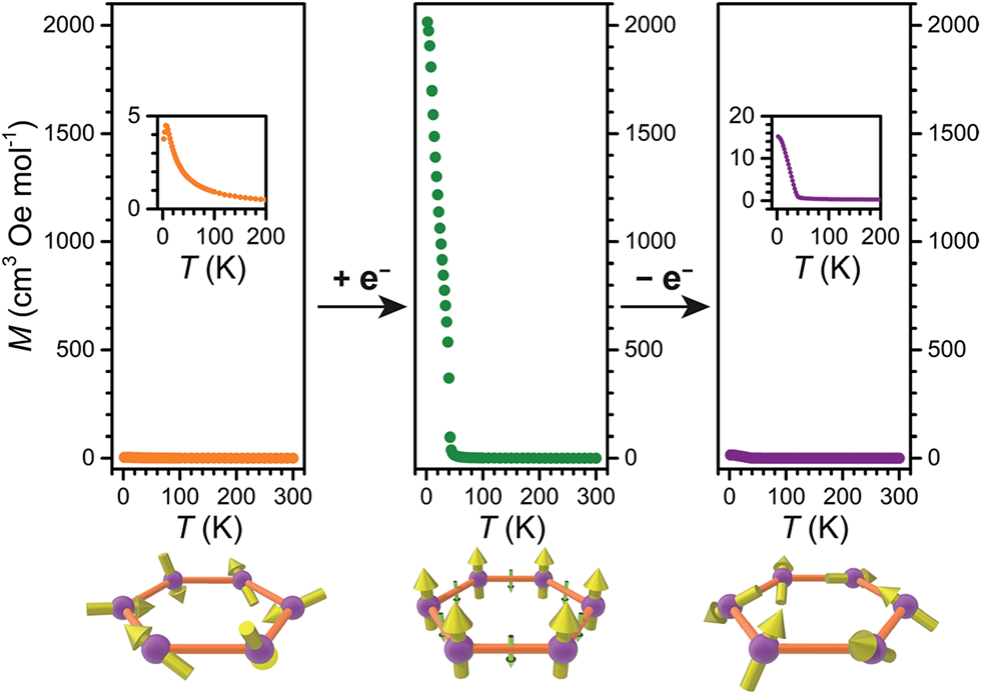
Variable-temperature field-cooled magnetization data for **2** (orange), **3** (green), and **4** (purple) collected under an applied dc field of 10 Oe. Insets show the data at a more easily visualized scale. Cartoons depict spin orientations of metal ion and linker, illustrating paramagnetism in **2** and **4** and magnetic order in **3**.

Long-range magnetic interactions stemming from the antiferromagnetic coupling between Mn^II^ and L^3–^˙ in **3** are clearly illustrated in the variable-temperature magnetization plot, obtained from data collected under an applied dc field of *H* = 10 Oe. As shown in Fig. 7, upon cooling from 300 K, the magnetization of **3** gradually increases, following a Curie–Weiss relationship to *ca.* 50 K (see Fig. S18†). Upon further cooling, an abrupt increase in the magnetization occurs, with the magnetization reaching a maximum value of *M* = 2018 cm^3^ Oe mol^–1^ at 1.8 K. Such an abrupt increase in magnetization is characteristic of long range magnetic order. Fitting the inverse magnetic susceptibility against temperature from 50 to 300 K using Curie–Weiss equation results in a Curie temperature of *θ* = 47 K and a Curie constant of *C* = 7.94 cm^3^ mol^–1^ K (see Fig. S18†). As further support of the spontaneous magnetization, a divergence in the magnetic susceptibility plot occurs below 40 K for **3** upon cooling the sample with and without an external dc field (see Fig. S19†).

**Fig. 8:**
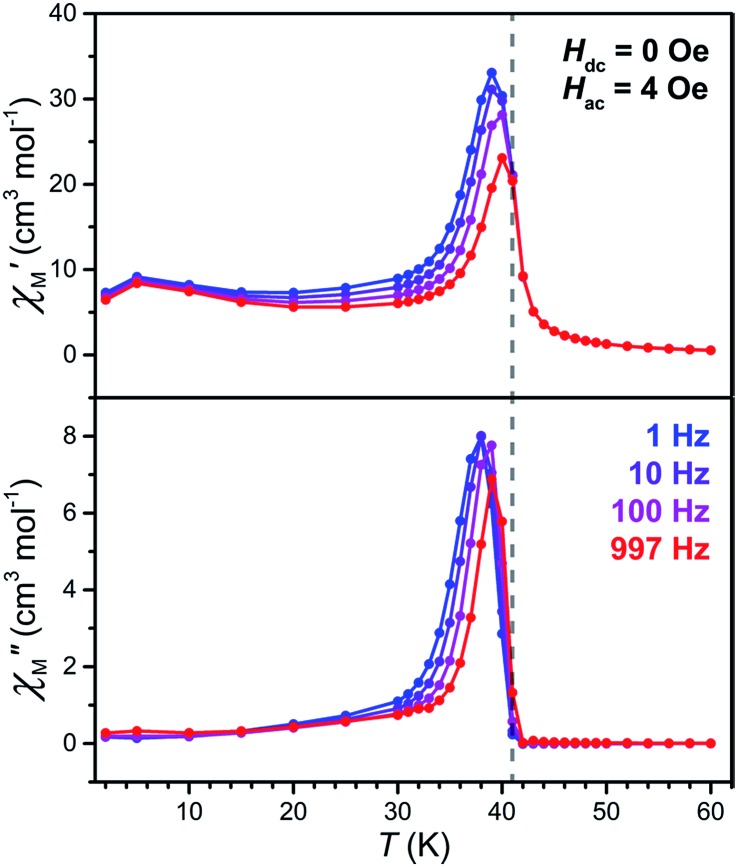
Variable-temperature in-phase (upper) and out-of-phase (lower) ac susceptibility data for **3**, collected under zero dc field at selected frequencies. The gray dashed vertical line serves as a visual guide and denotes an ordering temperature of *T*_c_ = 41 K.

To precisely determine the ordering temperature of **3**, variable-temperature ac magnetic susceptibility data were measured under zero applied dc field frequencies of 1, 10, 100, and 997 Hz. As shown in Fig. 8, an abrupt increase of both the in-phase (*χ*′M) and out-of-phase (*χ*′M) components of the ac susceptibility are observed below 42 K, establishing an ordering temperature of *T*_c_ = 41 K.11*n* A very minor frequency dependence is observed for the data, corresponding to a Mydosh parameter of *φ* = 0.0085, indicating the possible presence of some glassy behaviour or other complicated magnetization dynamics.^31^

**Fig. 9:**
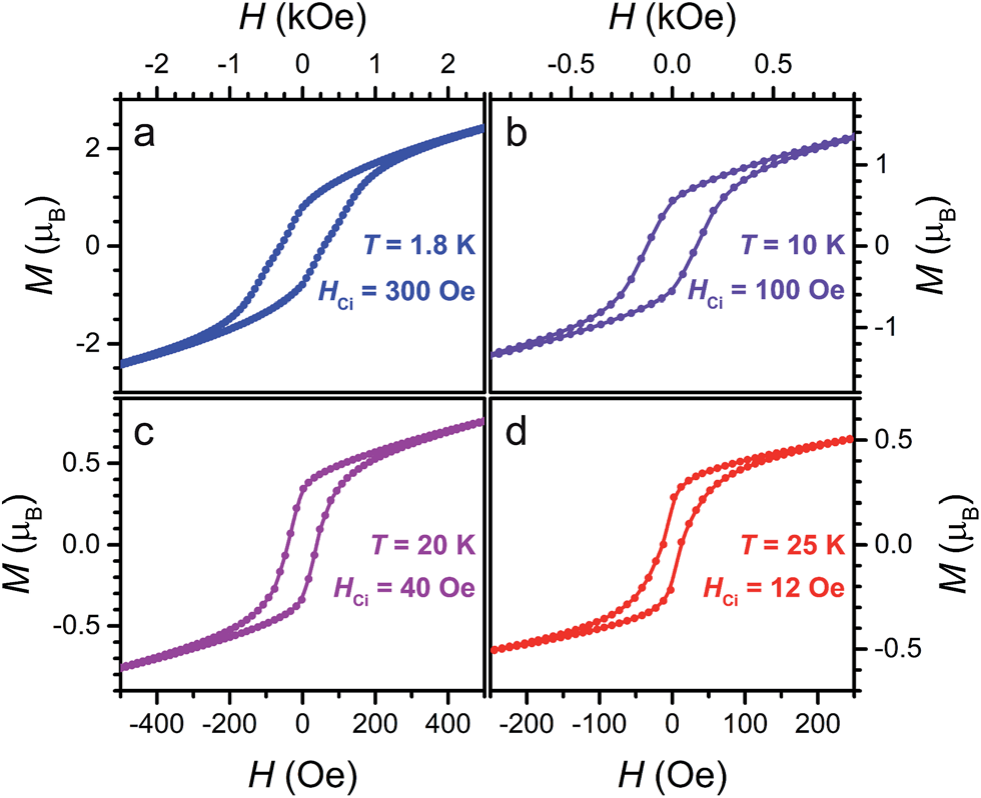
Variable-field magnetization data for **3** collected at 1.8, 10, 20, and 25 K, at sweep-rates of 0.6, 0.6, 0.2, and 0.1 Oe s^–1^, respectively.

Finally, the presence of magnetic hysteresis of **3** was probed by collecting variable-field magnetization data at selected temperatures. As shown in Fig. 9, open hysteresis loops are observed up to 25 K. Intrinsic coercive fields for **3** at 1.8, 10, 20 and 25 K were determined to be *H*_Ci_ = 300, 100, 40, and 12 Oe, respectively, with field-sweep rates of 0.6, 0.6, 0.2, and 0.1 Oe s^–1^. These *H*_Ci_ values of **3** are significantly lower than the iron analogue (Cp_2_Co^III^)_1.43_(Me_2_NH_2_)_1.57_[FeIII2(L^3–^˙)_3_], which features *H*_Ci_ = 4520 and 2270 Oe at 1.8 and 10 K, respectively. This difference likely stems from the negligible magnetic anisotropy associated with high-spin Mn^II^. Indeed, the behaviour of **3** as a soft magnet may render the compound useful in spintronics applications, where the direction of its spin polarization can be easily manipulated with a weak external magnetic field.1*b*

The ability to switch between a paramagnet down to 1.8 K and a permanent magnet is nearly unprecedented. In oxide-based dilute magnetic semiconductors or other 2D solid-state inorganic materials such as CrI_3_ and Fe_3_GeTe_2_, capacitive post-synthetic doping of charge carriers can tune ordering temperature and coercivity.^7^,8d,17c,d However, all of their redox states, which are accessed by modulation of an electric field, are nevertheless permanent magnets with different values of *T*_c_. Similarly, post-synthetic modulation of *T*_c_ has been demonstrated in the metal–organic permanent magnets (^*n*^Bu_4_N)[Cr^III^Mn^II^L_3_],^12^ (Me_2_NH_2_)_2_[Fe_2_L_3_],^19^ and (^*n*^Bu_4_N)_2_[Fe_2_(dhbq)_3_].^18^ Conversely, compound **2** is a paramagnet above 1.8 K, whereas the reduced compound **3** is a permanent magnet below *T*_c_ = 41 K, and this redox process is chemically reversible. To our knowledge, the only other MOF that can undergo reversible switching between paramagnetism and magnetic order is a Ru_2_ paddlewheel-TCNQ derivative.^13^ Compounds with similar simultaneous switching of magnetism and conductivity, albeit with higher magnetic ordering temperatures, may find use in future spintronic applications.^4^

## Summary and outlook

The foregoing results demonstrate the ability of metal–organic frameworks to undergo reversible redox-switching of magnetic order and electrical conductivity. The 2D manganese-benzoquinoid framework compound (Me_4_N)_2_[MnII2(L^2–^)_3_], accessed through SC–SC cation exchange, behaved as a paramagnet above 1.8 K with an ambient-temperature electrical conductivity of *σ*_295 K_ = 1.14(3) × 10^–13^ S cm^–1^ (*E*_a_ = 0.74(3) eV). Subsequent post-synthetic chemical reduction afforded the reduced semiquinoid compound Na_3_(Me_4_N)_2_[MnII2(L^3–^˙)_3_] *via* a SC–SC process. This latter species was found to be a permanent magnet with a characteristic temperature of *T*_c_ = 41 K, and its ambient-temperature conductivity of *σ*_295 K_ = 2.27(1) ×10^–8^ S cm^–1^ (*E*_a_ = 0.489(8) eV) represents a 200 000-fold increase relative to the oxidized form. Finally, the redox process was shown to be chemically reversible, with oxidation of the semiquinoid compound affording Na(Me_4_N)[MnII2(L^2–^)_3_]. Future efforts will target related compounds that undergo simultaneous switching of magnetism and conductivity, but with higher magnetic ordering temperatures and higher values of electrical conductivity.

## Conflicts of interest

There are no conflicts to declare.

## Supplementary Material

Supplementary information

Crystal structure data
