# Supplementary material for: Reversible redox switching of magnetic order and electrical conductivity in a 2D manganese benzoquinoid framework
Source: Chem Sci. 2019 Mar 14;10(17):4652–61. doi: 10.1039/c9sc00606k (PMC6495699; doi:10.1039/c9sc00606k)
Supplement: Supplementary file 1 [file SC-010-C9SC00606K-s001.pdf]

Electronic Supplementary Material (ESI) for Chemical Science.
This journal is © The Royal Society of Chemistry 2019

Electronic Supplementary Information for:

# Reversible Redox Switching of Magnetic Order and Electrical Conductivity in a 2D Manganese Benzoquinoid Framework

Lujia Liu, Jordan A. DeGayner, Lei Sun, David Z. Zee, and T. David Harris*

*Department of Chemistry, Northwestern University, 2145 Sheridan Road, Evanston IL 60208-3113, United States*

Email: dharris@northwestern.edu

*Chemical Science*

---

## Table of Contents

**Experimental Section** **S3**
**Table S1 |** Crystallographic data for **1-4** **S7**
**Figure S1 |** Experimental and simulated PXRD patterns of **2** **S8**
**Figure S2 |** $^1$H NMR spectrum of acid-digested **2** **S9**
**Figure S3 |** $^{13}$C NMR spectrum of acid-digested **2** **S10**
**Figure S4 |** Cyclic voltammogram of 1,2-dihydroacenaphthlyene **S11**
**Figure S5 |** $^1$H NMR spectrum of acid-digested **3** **S12**
**Figure S6 |** $^{13}$C NMR spectrum of acid-digested **3** **S13**
**Figure S7 |** $^1$H NMR spectrum of acid-digested **4** **S14**
**Figure S8 |** $^{13}$C NMR spectrum of acid-digested **4** **S15**
**Figure S9 |** $^1$H NMR spectrum of the supernatant of the oxidative conversion **S16**
**Figure S10 |** $^{13}$C NMR spectrum of the supernatant of the oxidative conversion **S17**
**Figure S11 |** A diffraction image and an optical microscopy image of **2** **S18**
**Figure S12 |** A diffraction image and an optical microscopy image of **3** **S19**
**Figure S13 |** A diffraction image and an optical microscopy image of **4** **S20**
**Figure S14 |** PXRD peak width analysis for **3** **S21**
**Figure S15 |** Variable-temperature field-cooled $\chi_M T$ data for **2** and **4** **S22**
**Figure S16 |** Variable-temperature $\chi_M T$ data for **3** **S23**
**Figure S17 |** Variable-field magnetization data for **3** **S24**

**Figure S18 |** Variable-temperature $1/\chi_M$ versus $T$ plot for **3** **S25**
**Figure S19 |** Variable-temperature $\chi_M$ data for **3** **S26**
**References** **S27**

## Experimental Section

**General Considerations.** Unless otherwise specified, chemicals and solvents were purchased from commercial vendors and used without further purification. Deuterated solvents were purchased from Cambridge Isotope Laboratories. Tetrahydrofuran (THF), acetonitrile, and diethyl ether ($Et_2O$) were dried using a commercial solvent purification system from Pure Process Technology and were stored over 3 or 4 Å molecular sieves prior to use. Water was obtained from a purification system from EMD Millipore. Elemental analysis was conducted by Midwest Microlab Inc. or in the Integrated Molecular Structure Education and Research Center (IMSERC) at Northwestern University. The compound $(Et_4N)_2[Mn_2L_3]\cdot x$DMF (**1**) was prepared using a previously described procedure.1

**Purification of 1,2-Dihydroacenaphthylene and Naphthalene.** In a dinitrogen-filled Vacuum Atmospheres Nexus II glovebox, 1,2-dihydroacenaphthlylene or sublimated naphthalene were dissolved in THF. The resulting solutions were stored over 4 Å molecular sieves for a minimum of 5 days at ambient temperature. Molecular sieves were removed by filtration, and 1,2-dihydroacenaphthlylene or naphthalene were collected by evaporating the THF under reduced pressure.

**NMR Spectroscopy.** For **2**–**4**, samples were digested using the following protocol. First, 23 µL of a 35% DCl solution in $D_2O$ was mixed with 1 mL of DMSO-$d_6$ to give a DCl/DMSO-$d_6$ stock solution. Approximately 2 mg of compound was then digested in 150 µL of this stock solution and 450 µL of DMSO-$d_6$. The reaction supernatant of **4** was concentrated by evaporating the solvent under reduced pressure to afford a free-flowing solid. This solid was dissolved in $CD_3CN$, and then the spectra were acquired. All $^1$H and $^{13}$C NMR spectra were acquired using a Bruker Avance III 500 MHz spectrometer equipped with a DCH CryoProbe.

**Inductively Coupled Plasma-Optical Emission Spectroscopy (ICP-OES).** To approximately 1–2 mg of crystalline **2**–**4**, concentrated nitric acid (0.5 mL) was added in a plastic falcon tube at 40 °C, followed by addition of one drop of $H_2O_2$ (30% aqueous solution). The resulting mixture was allowed to stand at 40 °C until no solid material remained (typically ca. 2 minutes). The resulting solution was diluted with Millipore $H_2O$ to 10 mL for ICP-OES analysis. The samples were analyzed using a Thermo Scientific iCAP 7600 instrument. The radial optical emission signal was acquired and analyzed. A blank sample and a set of standard solutions were measured immediately before measuring the samples. The software output the concentration of each metal in ppm, and this value was then converted to mole ratio.

**$(Me_4N)_2[Mn_2L_3]\cdot 3.2Et_2O$ (2).** The salt $(Me_4N)BF_4$ (362 mg, 2.25 mmol) was dissolved in DMF (15 mL) at 75 °C. The resulting solution was then added to crystals of $(Et_4N)_2[Mn_2L_3]\cdot x$DMF (**1**·$x$DMF, ca. 350 mg) suspended in DMF (0.5 mL), and the resulting mixture was allowed to stand at 75 °C. After 26 h, the colorless supernatant was decanted, and the remaining crystals were soaked in a fresh $(Me_4N)(BF_4)$ in DMF solution (0.15 M, 15 mL) at 75 °C for another 14 h. The resulting brown crystals were washed with DMF (6 × 5 mL) by iteratively decanting and replenishing fresh solvent, before they were collected by filtration and washed with $Et_2O$ (6 × 5 mL) to give **2** as dark brown hexagonal plate-shaped crystals (352 mg, ca. 98%). $^1$H NMR (acid-digested, 500 MHz, DCl/DMSO-$d_6$, see Figure S2): δ 3.90 (s, $H_3O^+$), 3.38 (q, $J$ = 6.9 Hz, $Et_2O$,

13H, overlaps with $H_3O^+$), 3.11 (s, $Me_4N^+$, 24H, overlaps with $H_3O^+$), 1.09 (t, $J$ = 6.9 Hz, $Et_2O$, 6.8H) ppm. $^{13}$C NMR (see Figure S3): δ 64.9 ($Et_2O$), 54.5 ($Me_4N^+$), 15.1 ($Et_2O$) ppm. The absence of the carbon peaks for $H_2L$ is likely due to the coordination of $Mn^{2+}$ in solution, as illustrated in Figure S3, where paramagnetic $Mn^{2+}$ ions induce fast nuclear relaxation of the proximal C atoms. Anal. Calcd for $C_{26}H_{24}Cl_6Mn_2N_2O_{12}\cdot 3.2(C_4H_{10}O)$: C, 41.75; H, 5.06; N, 2.51 %. Found: C, 41.45; H, 4.77; N, 2.09.

**$Na_3(Me_4N)_2[Mn_2L_3]\cdot 3.9THF$ (3).** In a dinitrogen-filled glovebox, crystals of **2** (247 mg, 221 mmol) were soaked in THF (5 mL) at ambient temperature for 1 minute. The supernatant was then decanted, and fresh THF (5 mL) was added. This decanting-replenishing process was repeated 5 times over the course of 24 h to afford a suspension in THF. In a separate vial, a solution of naphthalene (320 mg, 2.50 mmol) in THF (4 mL) was stirred over a sodium mirror at ambient temperature for 90 minutes. The resulting dark green solution was added dropwise to a solution of acenaphthene (382 mg, 2.48 mmol) in THF (2 mL). The resulting dark green solution was filtered through diatomaceous earth and was added to the suspension at ambient temperature. The resulting reaction mixture was then allowed to stand at −35 °C for 23 days. The reaction was then allowed to warm to ambient temperature, and the supernatant was decanted. The resulting crystals were washed with THF (10 × 5 mL) over a course of 24 h by iteratively decanting the supernatant and replenishing with fresh THF to give **3** as dark green hexagonal plate-shaped crystals (278 mg, 98%). ICP-OES: Na, 16.077 ppm; Mn, 25.702 ppm. This gives a mole ratio of Na:Mn = 2.99:2. $^1$H NMR (acid-digested, 500 MHz, DCl/DMSO-$d_6$, see Figure S5): δ 5.13 (s, $H_3O^+$), 3.60 (br, THF, 15.7H), 3.12 (s, $Me_4N^+$, 24H), 1.76 (br, THF, 14.4H) ppm. $^{13}$C NMR (see Figure S6): δ 165.3 ($H_2L$), 109.6 ($H_2L$) 67.0 (THF), 55.1 ($Me_4N^+$), 25.1 (THF) ppm. The appearance of the carbon peaks for $H_2L$ is likely due to the more acidic NMR solution compared to that of **2**, as evidenced by the relatively low-field $H_3O^+$ proton peak (see Figures S2 and S5). This might bias the (de)protonation-coordination equilibrium toward more $H_2L$ species, as illustrated in Figure S6. Anal. Calcd for $C_{26}H_{24}Cl_6Mn_2N_2Na_3O_{12}\cdot 3.9(C_4H_8O)$: C, 40.65; H, 4.53; N, 2.28 %. Found: C, 40.30; H, 4.91; N, 2.09.

**$Na(Me_4N)[Mn_2L_3]\cdot 5.5THF\cdot 0.8MeCN$ (4).** In a dinitrogen-filled glovebox, a solution of $(Cp_2Fe)(BF_4)$ (37.5 mg, 0.137 mmol) in a mixed solvent of THF/$CH_3CN$ (5 mL / 5 mL) was added to a polycrystalline solid sample of **3** (51 mg, 0.041 mmol). The reaction mixture was allowed to stand at room temperature for 24 h. The resulting pale green supernatant was decanted, collected separately for NMR study (see earlier), and replenished with fresh $CH_3CN$ (3 mL). This decanting-replenishing procedure was repeated three more times with $CH_3CN$ and one more time with THF (3 mL), to give **4** as dark brown hexagonal plate-shaped crystals (50 mg, 98%). ICP-OES: Na, 2.654 ppm; Mn, 12.757 ppm. This gives a mole ratio of Na : Mn = 1.00 : 2. $^1$H NMR (acid-digested, 500 MHz, DCl/DMSO-$d_6$, see Figure S7): δ 4.08 (s, $H_3O^+$), 3.60 (br, THF), 3.11 (s, $Me_4N^+$), 2.09 (s, $CH_3CN$), 1.76 (br, THF) ppm. $^{13}$C NMR (see Figure S8): δ 67.0 (THF), 54.7 ($Me_4N^+$), 25.1 (THF) ppm. Anal. Calcd for $C_{22}H_{12}Cl_6Mn_2NNaO_{12}\cdot 5.5(C_4H_8O)\cdot 0.8(C_2H_3N)$: C, 43.56; H, 4.68; N, 2.01 %. Found: C, 43.30; H, 4.94; N, 2.08.

**Cyclic Voltammetry.** Approximately 1 mg of 1,2-dihydroacenaphthylene was dissolved in a 0.1 M solution of $(^nBu_4N)PF_6$ in THF. This solution was analyzed at a scan rate of 100 mV/s in a

standard one-compartment cell under dinitrogen, equipped with a glassy carbon disk working electrode (3 mm diameter), a platinum wire as the counter electrode, and a silver wire as the reference electrode, using a CHI 660E potentiostat. Ferrocene was used as an internal standard and all potentials are referenced to the $[Cp_2Fe]^{+/0}$ couple.

**Single-Crystal X-Ray Structure Determination.** For **2** and **4**, the crystals were immersed in DMF to keep the compounds solvated before they were coated with a small amount of Paratone oil for data collection. A crystal of **3** was coated with deoxygenated Paratone oil in a dinitrogen-filled glovebox to minimize the exposure to the ambient air during mounting the crystal. Data collections were performed on a Bruker Kappa Apex II diffractometers, which was equipped with a Mo Kα sealed tube source, a graphite monochromator and a Bruker Kappa APEX CCD area detector. Suitable MOF single crystals were coated with Paratone-N oil and mounted on a MicroMounts or a MiTeGen rod. The data collections were performed at 250 K. Raw data were integrated, scaled and averaged using the Bruker APEX3 software.[2] Absorption corrections were applied using SADABS.[3] Space groups were determined by examination of systematic absence, *E*-statistics using XPREP[4] and successive refinement of structures. Structures were solved using SHELXT[5] and refined with SHELXL[5] using the Olex 2 graphical interface.[6] All framework atoms were solved and refined anisotropically. For the structures of **3** and **4**, sodium ions were not found in the Fourier difference map and therefore were not modeled. These sodium ions and occluded solvent molecules in **2**–**4** are not included in the formulae determined by crystallography. To finalize the structure refinements, residual electron densities contributed from disordered solvent molecules and counterions were removed using the solvent mask protocol in Olex 2.[6] The total electron counts per unit cell were 190 for **2** (2.60 DMF), 159 for **3** (3 Na and 1.25 THF), and 220 for **4** (1 Na and 2.70 DMF). Crystallographic data and the details of data collection are listed in Table S1.

**Powder X-ray Diffraction Analyses.** Approximately 1 mg of polycrystalline samples where loaded in a hollow metallic sample holder and both sides sealed with Kapton tape. For **1**, **2**, and **4**, a small amount of DMF was added to the sample to ensure the samples were kept fully-solvated during the measurement. For **3**, a sample was prepared in a dinitrogen-filled glovebox, without addition of DMF. The diffraction patterns were collected at a transmission geometry with a $2\theta$ step size of 0.015° using a STOE STADI MP instrument equipped with a Cu Kα1 sealed tube source and a 1D strip detector covering $2\theta$ range of 6°. The sample holder was spun during the measurement to obtain a better powdered-average. Unit cell parameters were obtained by indexing the peak positions followed by the cell refinement using the WinXPOW software package.[7] Simulated PXRD patterns were obtained from their single crystal structures.

**Raman Spectroscopy.** Crystals of **1**–**4** were deposited onto a silicon oxide-coated silicon wafer and sealed in a Linkam THMS350 V microscope stage. Raman spectra were collected using a Horiba LabRam HR Evolution confocal microscope. Individual crystals were excited with a 473 nm continuous-wave diode laser at 0.236 mW power equipped with a long working distance 50× microscope objective (NA = 0.50; Nikon) and 600 grooves/mm grating.

**Electrical Conductivity Measurements at 295 K.** Two-point dc conductivities for **2**–**4** were measured in a dinitrogen-filled glovebox at 295 K using a home-built press[8] and a CH Instruments

CHI660E Electrochemical Workstation. Polycrystalline samples were pressed between two steel rods of 2.94 mm diameter inside a glass tube. The thickness of the pressed pellets, measured by calipers, range from 0.2 to 0.8 mm. The applied bias across the pellets ranges between –0.1 and +0.1 V with a step size of 0.005 V, and current was measured at each step. The error of the conductivity is determined by the accuracy of the pellet length, or the slope error of the linear regression, whichever is larger.

**Variable-Temperature Electrical Conductivity Measurements.** Variable-temperature two-point dc conductivity for **2** and **3** were measured at temperature ranges of 311 to 382 K, and 275 to 375 K, respectively. Polycrystalline samples were pressed in a dinitrogen-filled glovebox using a home-built two-screw press[9] before being attached to a SQUID sample rod using silver epoxy. The sample rod was then lowered into the cryostat-equipped SQUID chamber, and data were collected using a CH Instruments CHI660E Electrochemical Workstation. I-V curves were measured multiple times after the temperature equilibrated to obtain multiple conductivity values. Mean values and standard deviations were obtained based on ln $\sigma$. The temperature-dependent conductivity behavior was found to follow an Arrhenius relationship:

$$\sigma = \sigma_0 \exp(-\frac{E_a}{k_B T})$$

where $\sigma_0$ is a prefactor, $E_a$ is the activation energy, $k_B$ is the Boltzmann constant, and $T$ is the absolute temperature. The error of the activation energy is determined by the accuracy of the pellet length, or the slope error of the linear regression, whichever is larger.

**Magnetic Measurements.** Magnetic measurements of **2**, **3**, and **4** were carried out on polycrystalline samples sealed in a polyethylene bag in a dinitrogen-filled glovebox. All data were collected using a Quantum Design MPMS-XL SQUID magnetometer from 1.8 to 300 K at applied dc fields ranging from 0 to 7 T. Ac magnetic susceptibility data under a field of 4 Oe oscillating at frequencies ranging from 1 to 997 Hz. Dc susceptibility data were corrected for diamagnetic contributions from the sample holder and for the core diamagnetism of each sample (estimated using Pascal's constants).[10] The coherence of the collected data was confirmed across multiple measurements. The magnetic ordering temperature for **3** was determined by the temperature below which a steep increase in $\chi_M''$ occurs upon cooling.

**Table S1 |** Crystallographic data for MOFs.

| Compound | $(Me_4N)_2[Mn_2L_3]\cdot 3.2Et_2O$ (**2**) | $Na_3(Me_4N)_2[Mn_2L_3]\cdot 3.9THF$ (**3**) | $Na(Me_4N)[Mn_2L_3]\cdot 5.5THF\cdot 0.8CH_3CN$ (**4**) |
|---|---|---|---|
| Formula based on crystallography | $C_{26}H_{24}Cl_6Mn_2N_2O_{12}$ | $C_{26}H_{24}Cl_6Mn_2N_2O_{12}$ | $C_{22}H_{12}Cl_6Mn_2NO_{12}$ |
| Formula weight | 879.09 | 879.09 | 804.94 |
| Temperature/K | 250(1) | 250(1) | 250(1) |
| Crystal system | trigonal | trigonal | trigonal |
| Space group | *P*-31*m* | *P*-31*m* | *P*-31*m* |
| *a, b*/Å | 14.0339(11) | 14.030(3) | 14.0833(13) |
| *c*/Å | 10.0156(8) | 9.3030(19) | 10.0253(12) |
| Volume/Å$^3$ | 1708.3(3) | 1585.9(3) | 1722.0(4) |
| Z | 1 | 1 | 1 |
| $\rho_{calc}$g/cm$^3$ | 0.854 | 0.920 | 0.776 |
| μ/mm$^{-1}$ | 0.635 | 0.684 | 0.626 |
| F(000) | 442.0 | 442.0 | 399.0 |
| Crystal size/mm$^3$ | 0.223 × 0.186 × 0.057 | 0.151 × 0.129 × 0.073 | 0.113 × 0.087 × 0.027 |
| Radiation | MoKα (λ = 0.71073) | MoKα (λ = 0.71073) | MoKα (λ = 0.71073) |
| 2Θ range | 3.352 to 52.838 | 3.352 to 37.788 | 5.26 to 46.496 |
| Index ranges | -17 ≤ h ≤ 17<br>-13 ≤ k ≤ 17<br>-12 ≤ l ≤ 12 | -12 ≤ h ≤ 12<br>-12 ≤ k ≤ 12<br>-8 ≤ l ≤ 8 | -13 ≤ h ≤ 13<br>-15 ≤ k ≤ 15<br>-11 ≤ l ≤ 11 |
| Total/independent reflections | 24027/1278 | 4242/469 | 13161/897 |
| Data/restraints/parameters | 1278/15/47 | 469/18/47 | 897/18/47 |
| Goodness-of-fit | 1.126 | 1.902 | 1.518 |
| Final R indexes [I>=2σ (I)] | $R_1$ = 0.0750; w$R_2$ = 0.2574 | $R_1$ = 0.1530; w$R_2$ = 0.4108 | $R_1$ = 0.1305; w$R_2$ = 0.3529 |
| Final R indexes [all data] | $R_1$ = 0.0863; w$R_2$ = 0.2731 | $R_1$ = 0.1681; w$R_2$ = 0.4251 | $R_1$ = 0.1725; w$R_2$ = 0.4018 |
| Largest diff. peak/hole / e Å$^{-3}$ | 0.64/-0.53 | 0.91/-0.66 | 1.54/-0.58 |

[a] $R_1 = \Sigma||F_0| - |F_C||/\Sigma|F_0|$

[b] $wR_2 = [\Sigma w(F_0^2 - F_C^2)^2/\Sigma w(F_o^2)^2]^{1/2}$

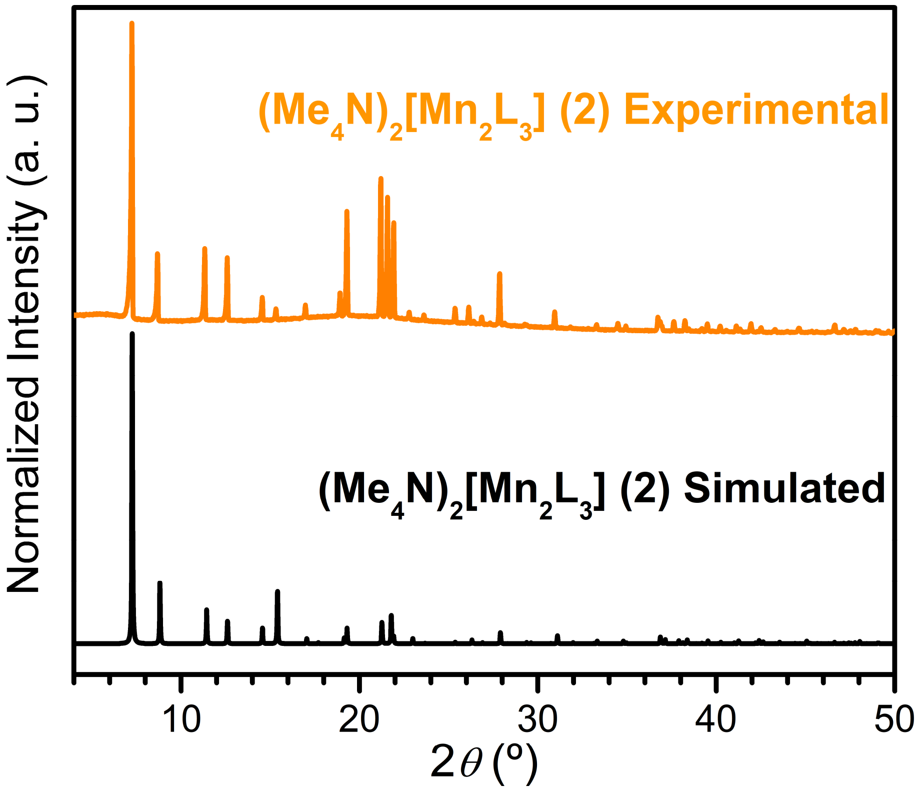


**Figure S1.** Experimental and simulated PXRD patterns of **2**.

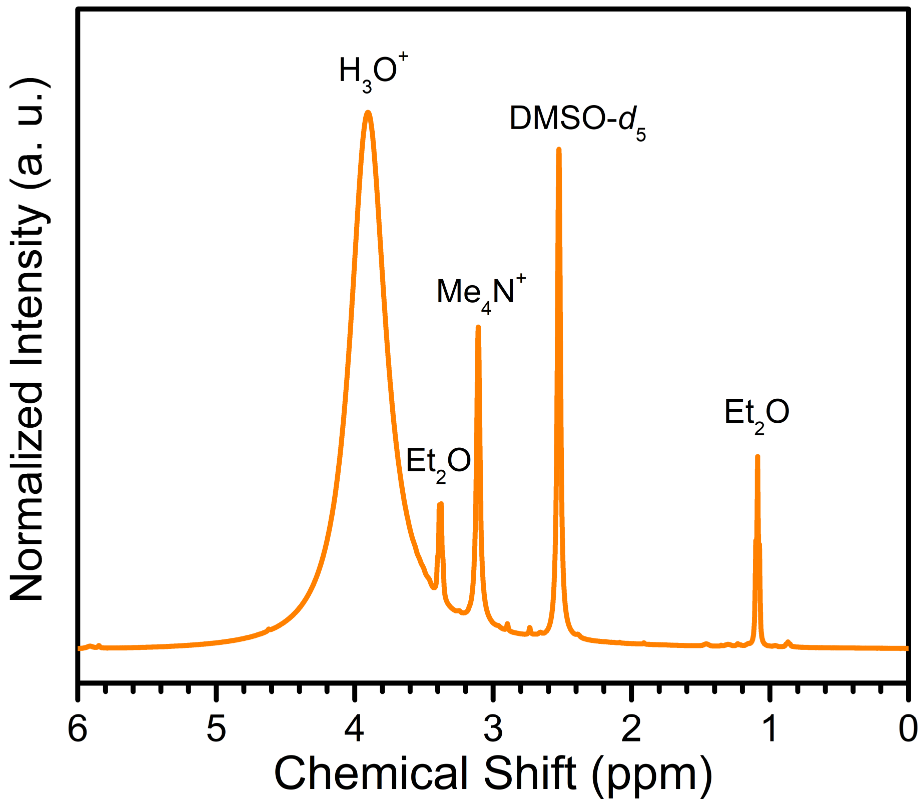


**Figure S2.** $^1$H NMR spectrum of an acid-digested sample of **2**.

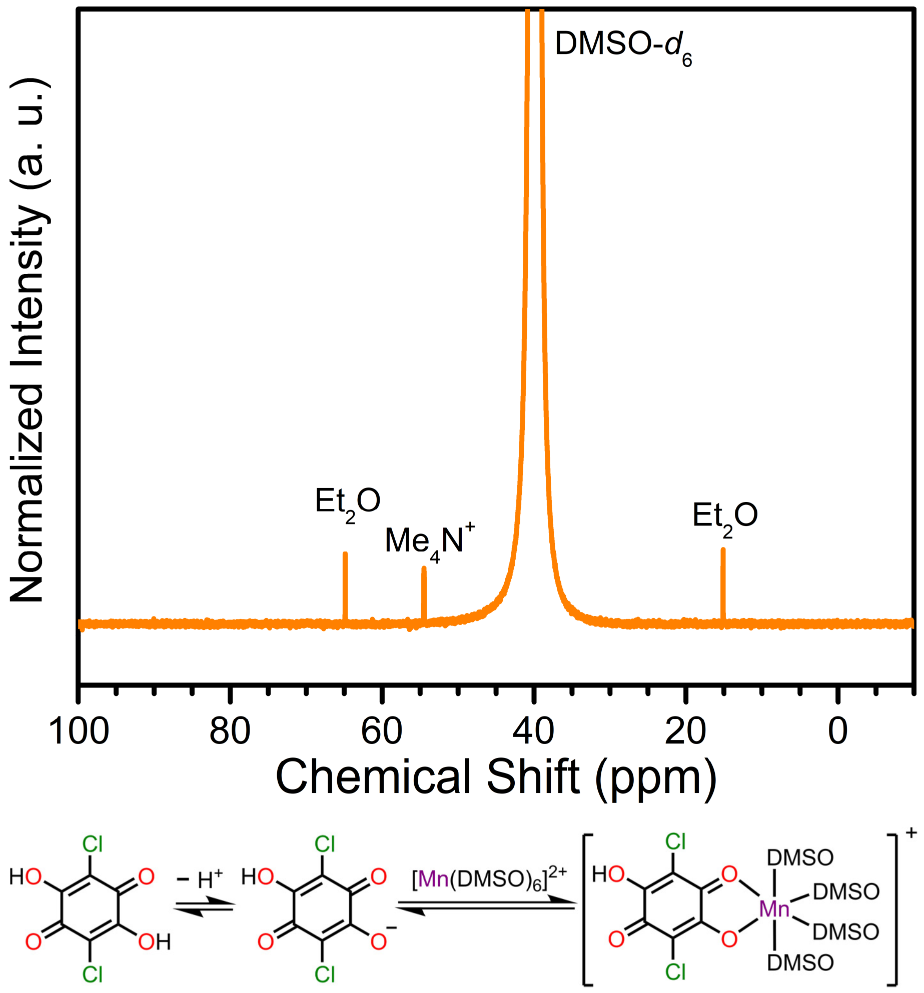


**Figure S3.** Top: $^{13}$C NMR spectrum of an acid-digested sample of **2**. Bottom: a proposed mechanism showing the dynamic deprotonation and a subsequent coordination of $H_2L$, which likely caused the significant broadening of the peaks due to the paramagnetic $Mn^{2+}$ ions that induce fast nuclear relaxation for the carbon atoms in its proximity.

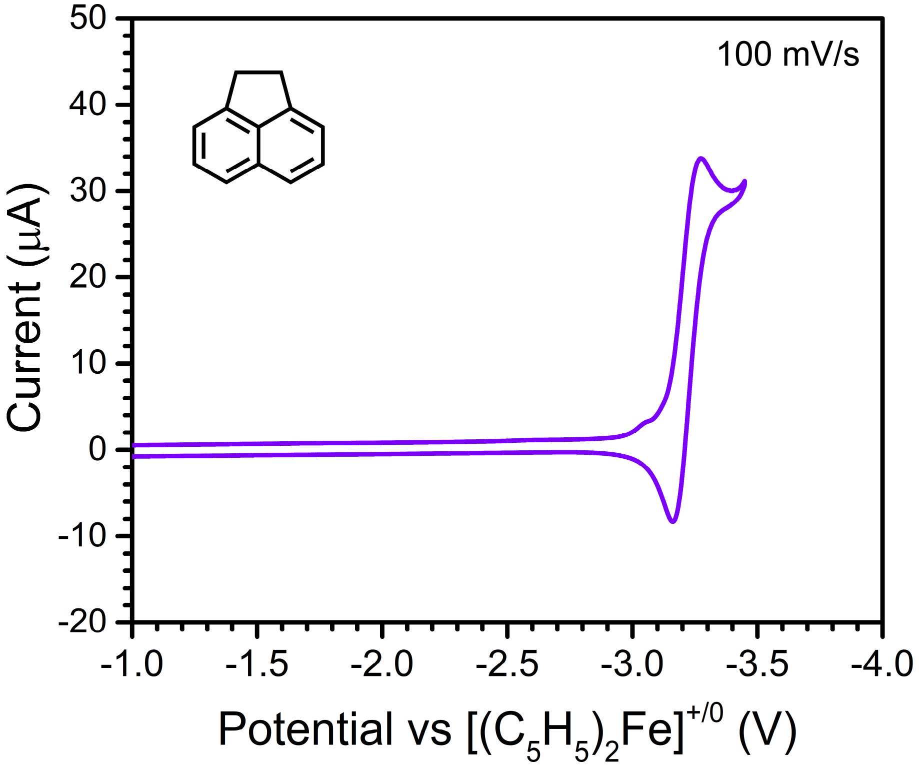


**Figure S4.** Cyclic voltammogram of 1,2-dihydroacenaphthlyene obtained in a 0.1 M ($^n$Bu$_4$N)PF$_6$ solution in THF.

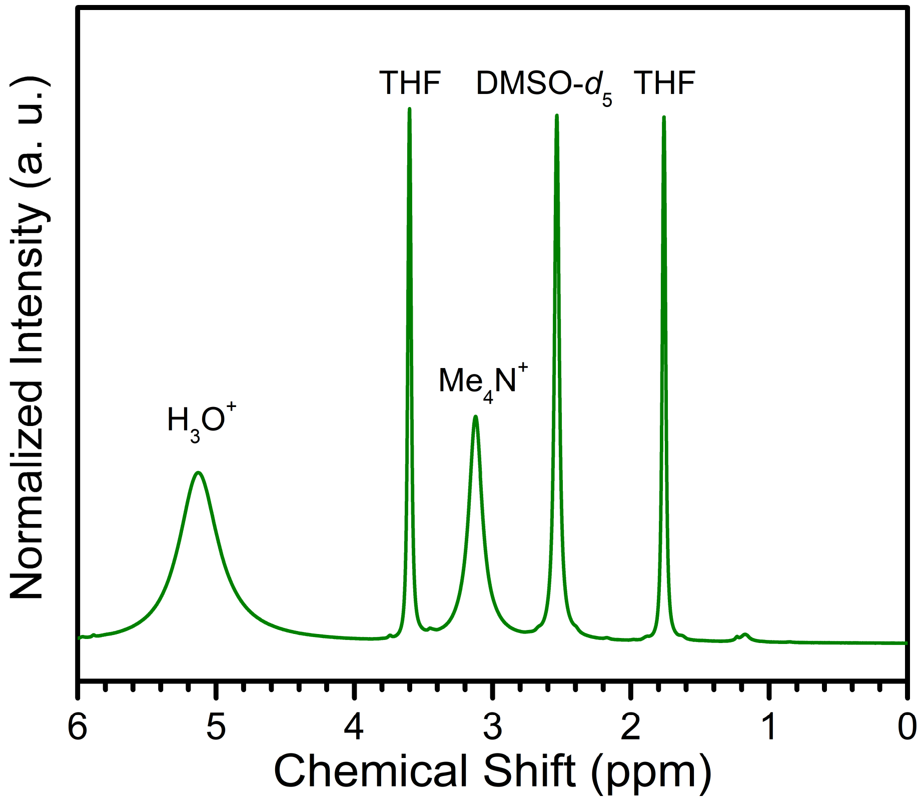


**Figure S5.** $^{1}H$ NMR spectrum of an acid-digested sample of **3**.

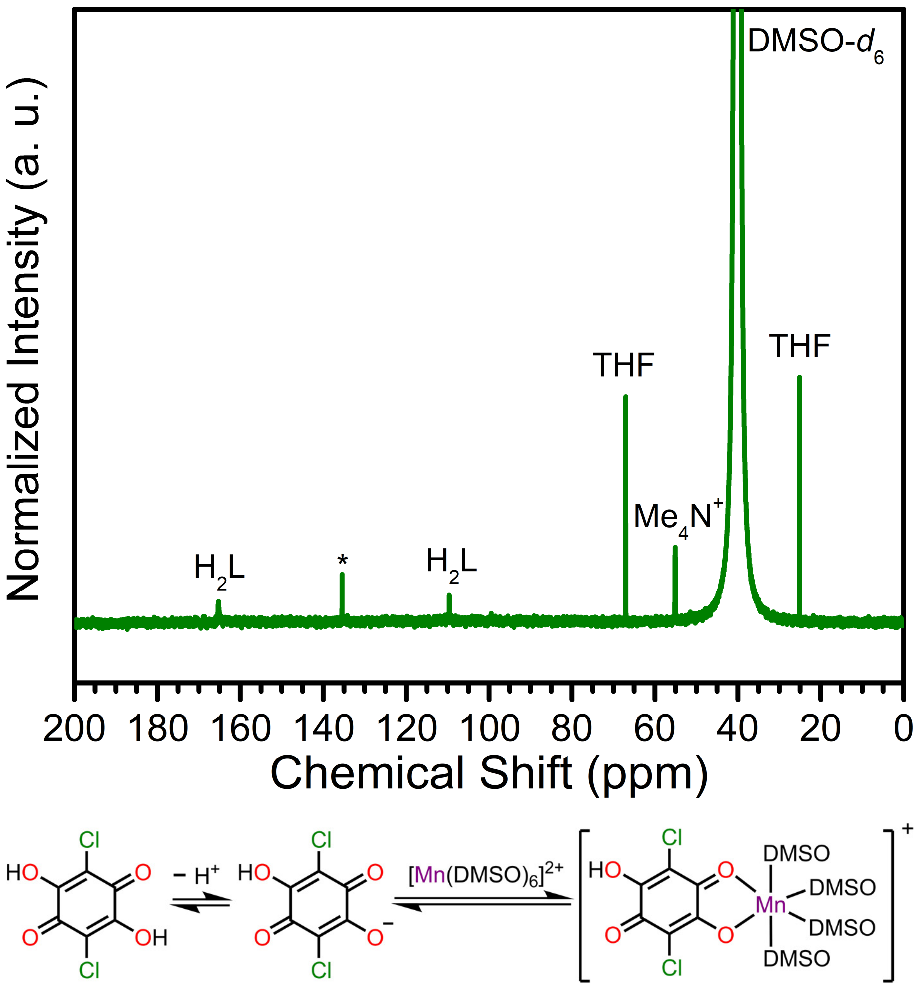


**Figure S6.** Top: $^{13}C$ NMR spectrum of an acid-digested sample of **3**. Asterisk denotes impurity. Bottom: a proposed mechanism showing the dynamic deprotonation and a subsequent coordination of $H_2L$. According to the $^1H$ NMR spectrum of **3** (see Figure S5), the chemical shift of $H_3O^+$ is at lower field compared to that of **2** (see Figure S2), suggesting this NMR solution is more acidic. This likely pushes the equilibrium towards the left and hence the ligand carbon peaks appear.

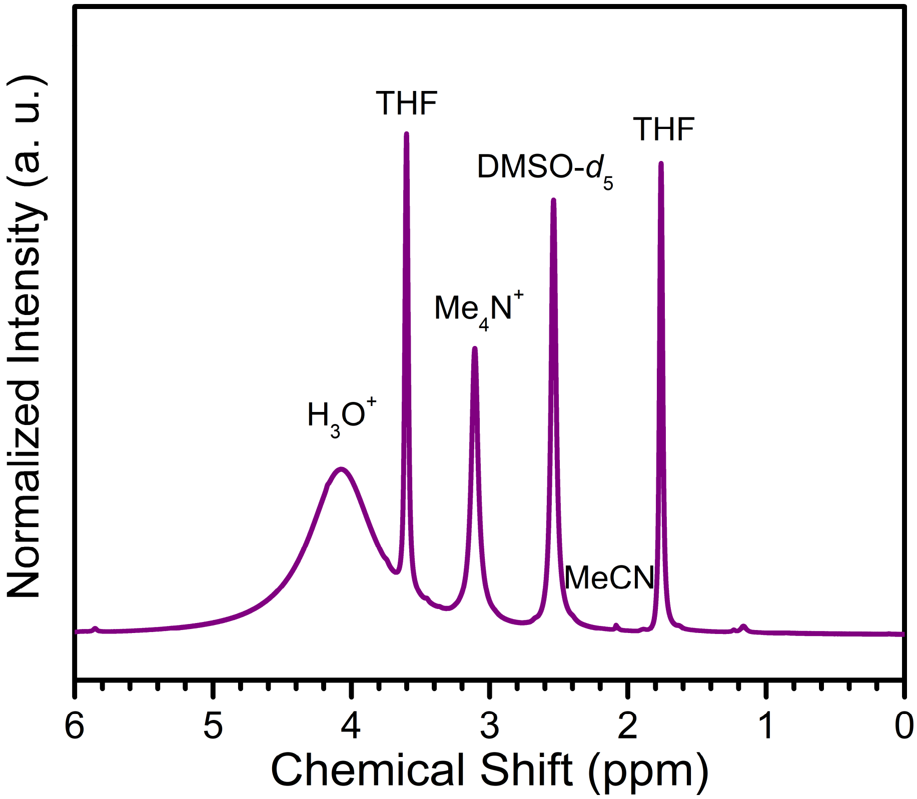


**Figure S7.** $^1$H NMR spectrum of an acid-digested sample of **4**.

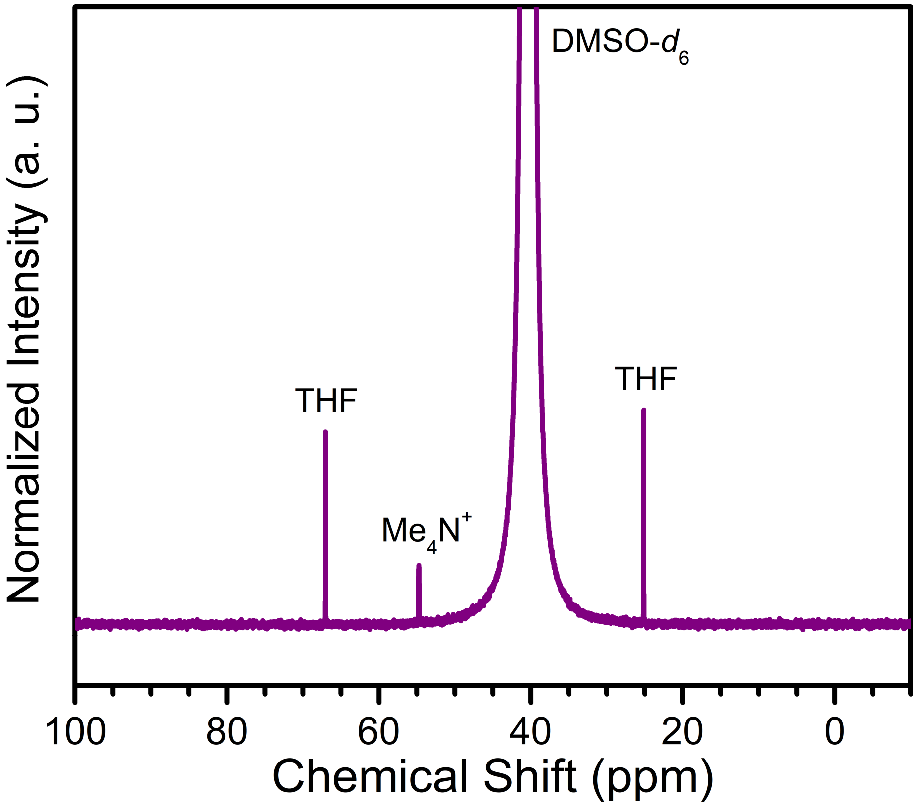


**Figure S8.** $^{13}C$ NMR spectrum of an acid-digested sample of **4**.

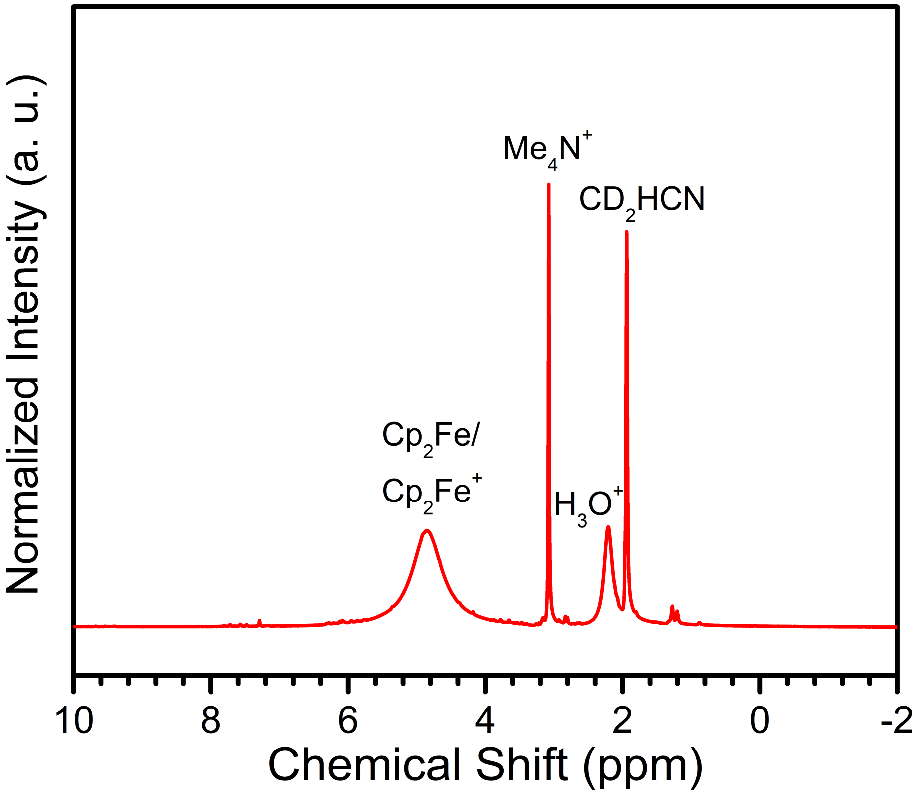


**Figure S9.** $^{1}$H NMR spectrum of the reaction supernatant for the oxidative conversion from **3** to **4**, with its solvent (THF/$CH_3CN$) evaporated under vacuum prior to the acquisition of the spectrum using $CD_3CN$.

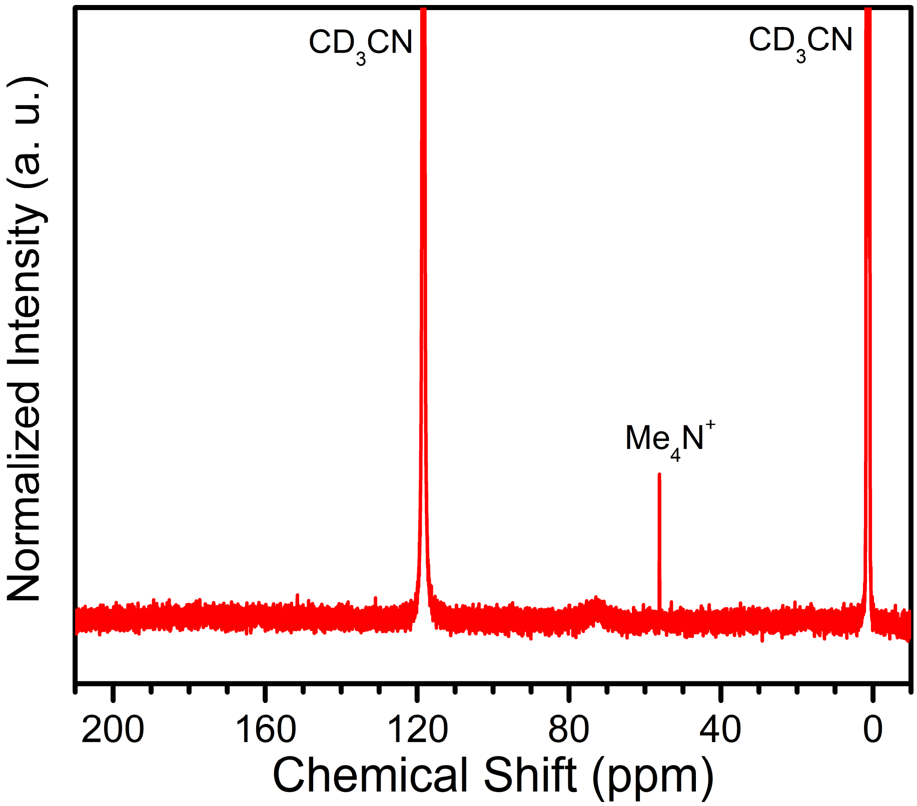


**Figure S10.** $^{13}C$ NMR spectrum of the reaction supernatant for the oxidative conversion from **3** to **4**, with its solvent (THF/$CH_3CN$) evaporated under vacuum prior to the acquisition of the spectrum using $CD_3CN$.

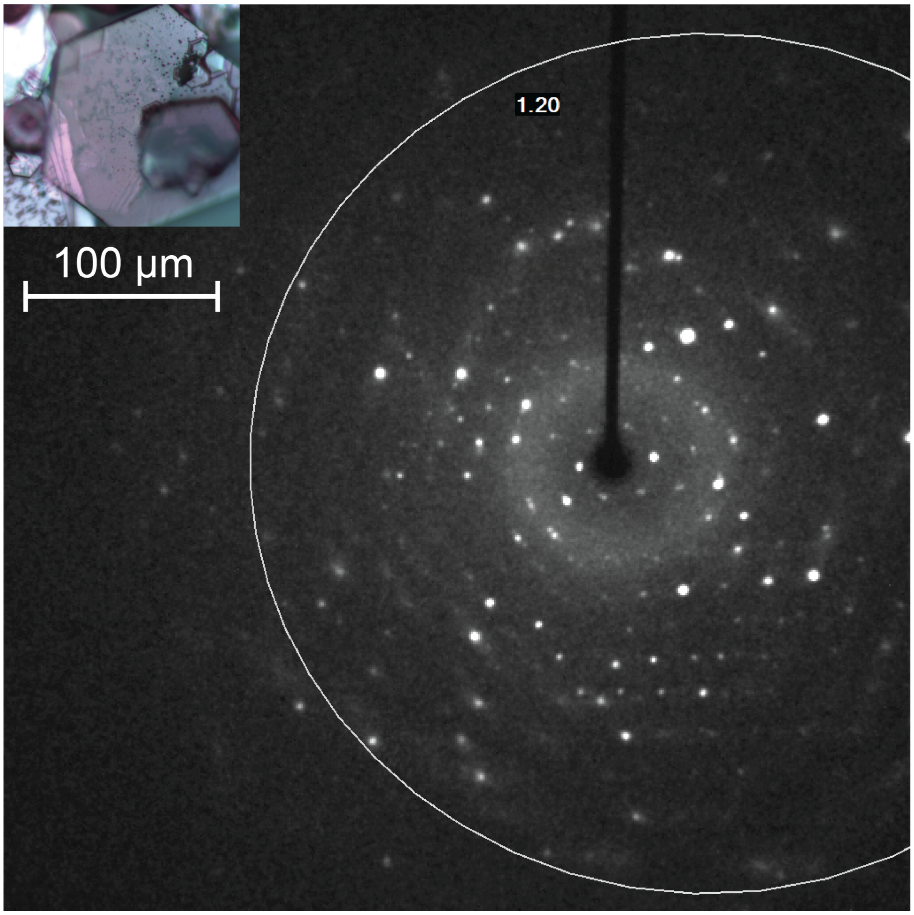


**Figure S11.** A diffraction image of **2**. The circle indicates a shell with a resolution of 1.2 Å. Inset: an optical microscopy image of crystals of **2**.

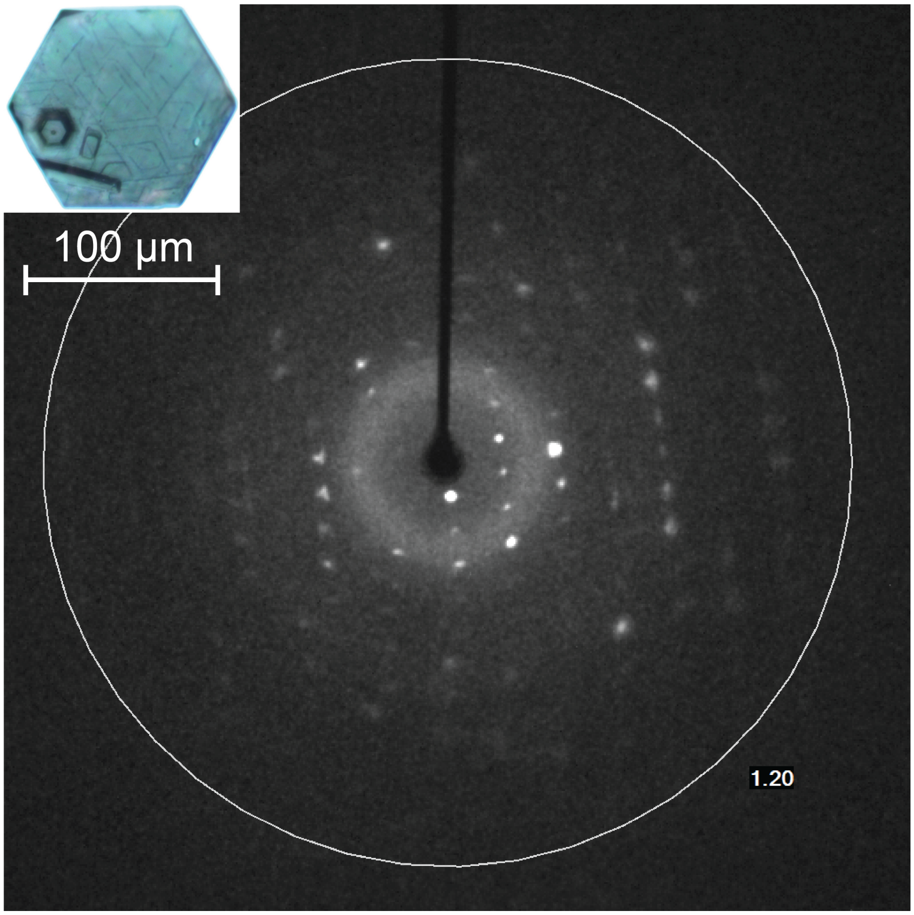


**Figure S12.** A diffraction image of **3**. The circle indicates a shell with a resolution of 1.2 Å. Inset: an optical microscopy image of a crystal of **3**.

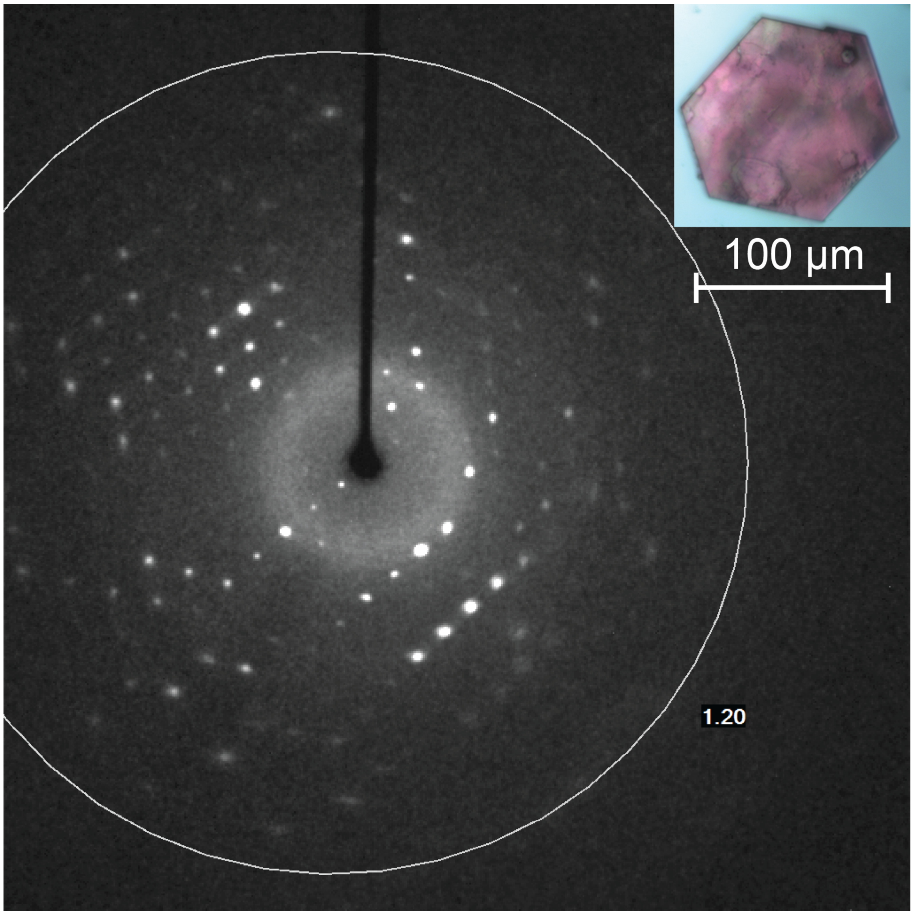


**Figure S13.** A diffraction image of **4**. The circle indicates a shell with a resolution of 1.2 Å. Inset: an optical microscopy image of a crystal of **4**.

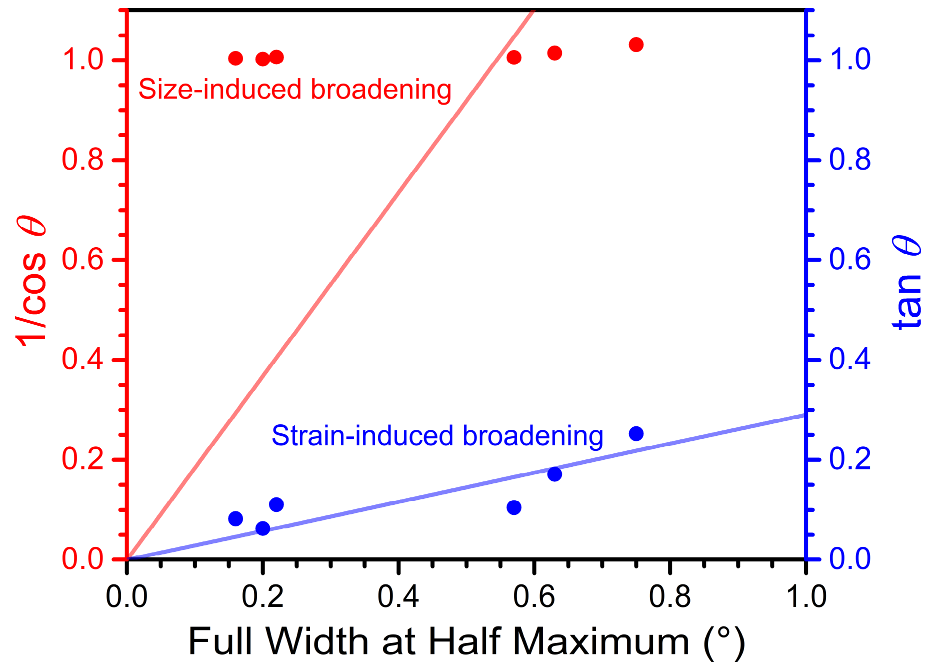


**Figure S14.** PXRD peak width analysis of **3**. Peak widths are plotted against 1/cos $\theta$ (red) and tan $\theta$ (blue), respectively. Red and blue lines indicate linear regression fits with intercepts equal to zero, according to Scherrer equation [$\beta_L = 0.9\ \lambda / (L \cos \theta)$; where $\beta_L$ is size-induced broadening, $\lambda$ is the X-ray wavelength, and $L$ is the crystallite size] and strain equation [$\beta_\varepsilon = 4\ \varepsilon \tan \theta$; where $\beta_\varepsilon$ is strain-induced broadening, and $\varepsilon$ is strain].

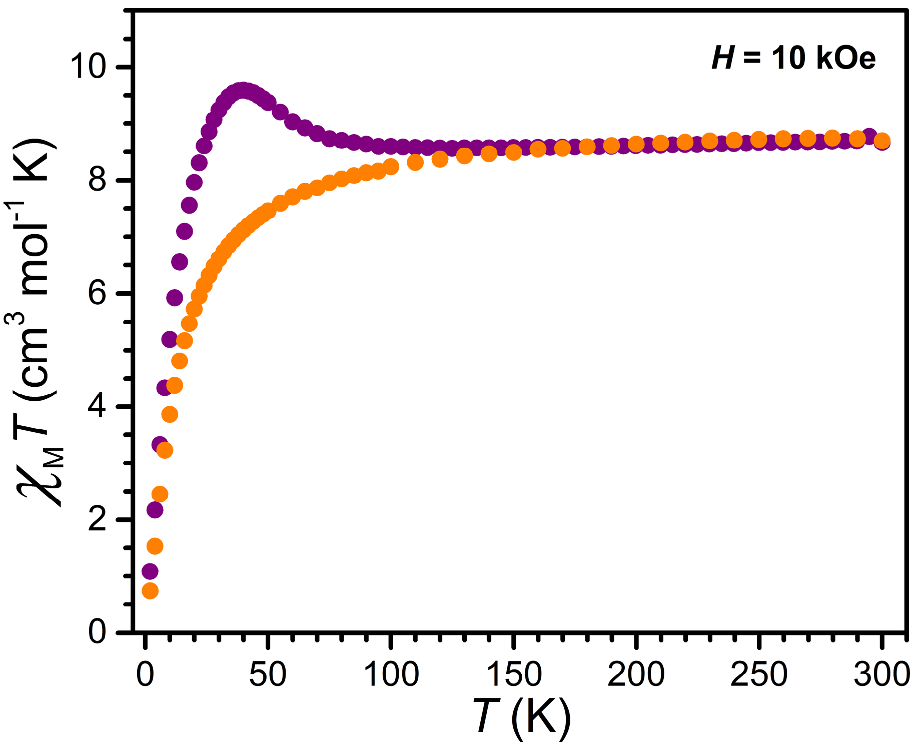


**Figure S15.** Variable-temperature field-cooled $\chi_M T$ data collected for **2** (orange) and **4** (purple) under applied dc fields of 10 kOe.

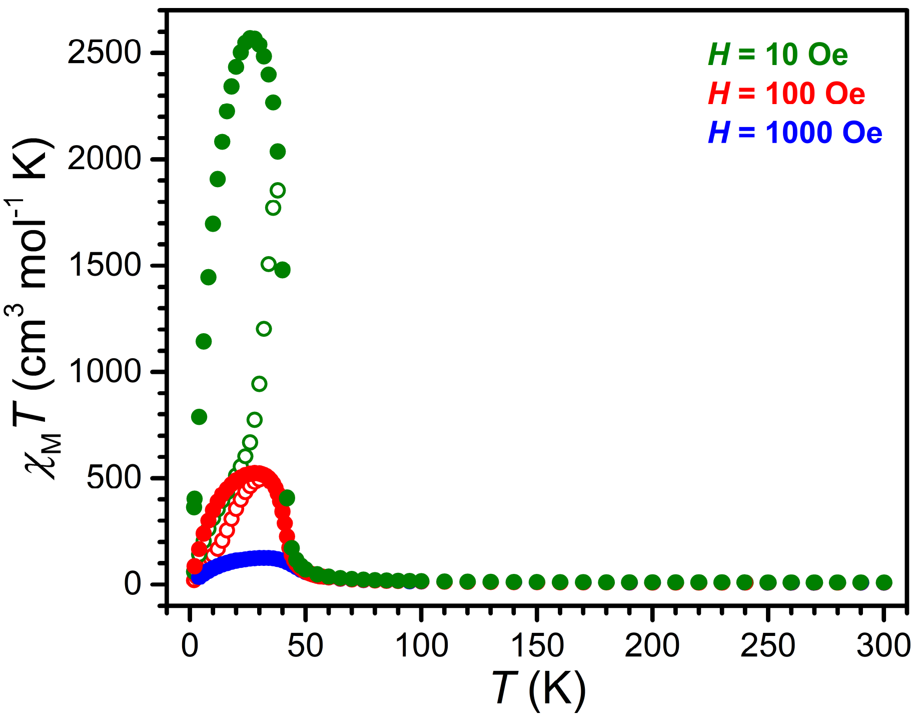


**Figure S16.** Variable-temperature zero-field-cooled (open circles) and field-cooled (closed circles) $\chi_M T$ data collected for **3** under applied dc fields of 10 (dark green), 100 (red), and 1000 (blue) Oe.

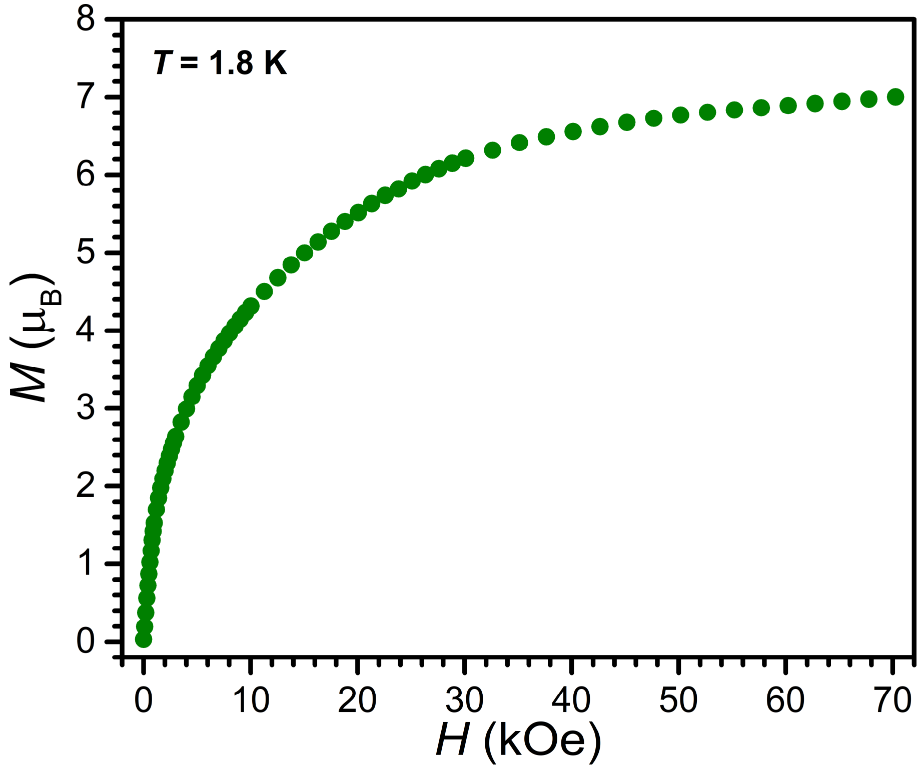


**Figure S17.** Variable-field magnetization data for **3** collected at 1.8 K.

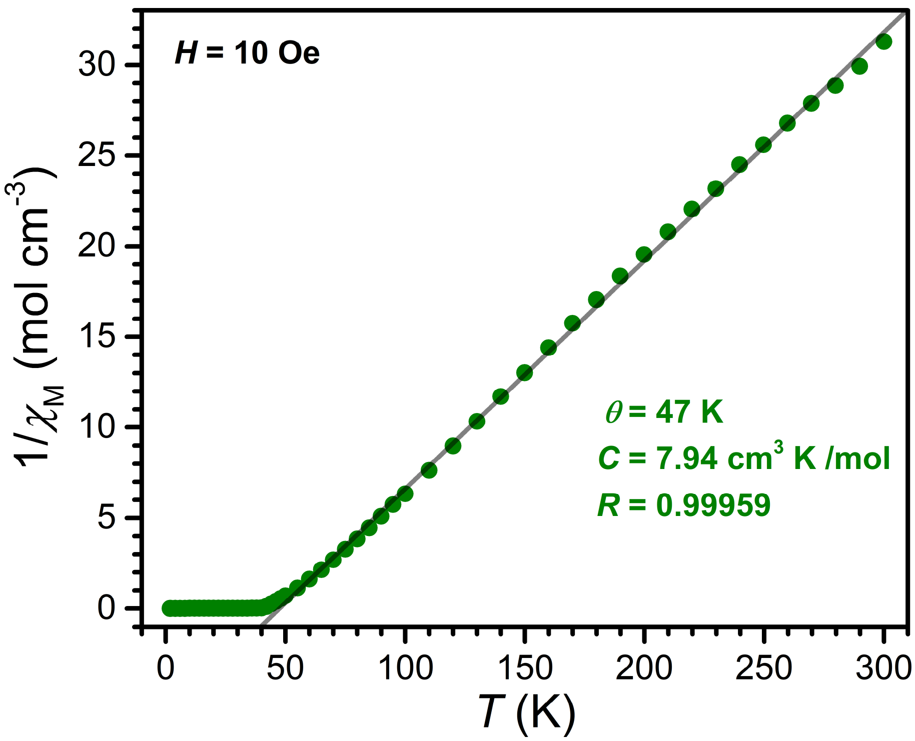


**Figure S18.** Variable-temperature field-cooled $1/\chi_M$ versus $T$ plot for **3** under applied dc fields of 10 Oe. The gray line represents the fitting using Curie-Wiess equation within the temperature range between 50 and 300 K.

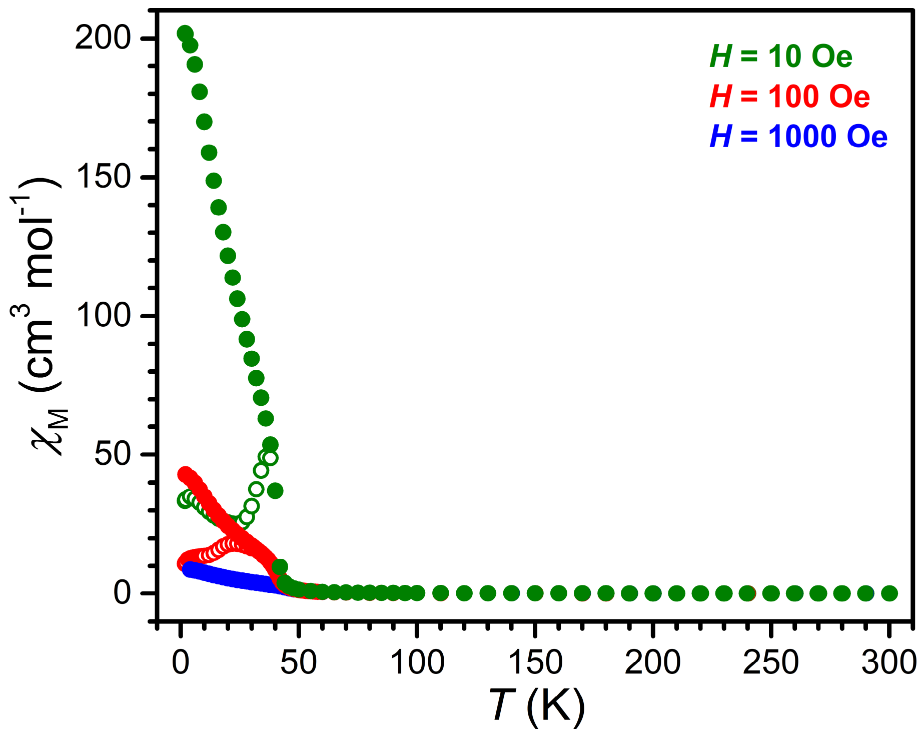


**Figure S19.** Variable-temperature zero-field-cooled (open circles) and field-cooled (closed circles) $\chi_M$ data collected for **3** under applied dc fields of 10 (dark green), 100 (red), and 1000 (blue) Oe.

**References**


1. L. Liu, L. Li, J. A. DeGayner, P. H. Winegar, Y. Fang and T. D. Harris, *J. Am. Chem. Soc.*, 2018, **140**, 11444-11453.

2. APEX 3, v. 2016.1-0; Bruker Analytical X-Ray Systems, Inc: Madison, WI, 2016.

3. Sheldrick, G. M. SADABS, version 2.03, Bruker Analytical X-Ray Systems, Madison, WI, 2000

4. Sheldrick, G.M. XPREP, version 2008/2, Bruker Analytical X-Ray Systems, Inc., Madison, WI, 2008.

5. G. Sheldrick, *Acta Crystallogr. A*, 2008, **64**, 112-122.

6. O. V. Dolomanov, L. J. Bourhis, R. J. Gildea, J. A. K. Howard and H. Puschmann, *J. Appl. Crystallogr.*, 2009, **42**, 339-341.

7. WINXPOW Powder Diffraction Software (v3.5.0.1), STOE & Cie GmbH, Hilpertstr. 10, 64295 Darmstadt, Germany, 2017.

8. L. Sun, S. S. Park, D. Sheberla and M. Dincă, *J. Am. Chem. Soc.*, 2016, **138**, 14772-14782.

9. L. E. Darago, M. L. Aubrey, C. J. Yu, M. I. Gonzalez and J. R. Long, *J. Am. Chem. Soc.*, 2015, **137**, 15703-15711.

10. G. A. Bain and J. F. Berry, *J. Chem. Educ.*, 2008, **85**, 532.
